# PreImplantation factor (PIF*) promotes embryotrophic and neuroprotective decidual genes: effect negated by epidermal growth factor

**DOI:** 10.1186/1866-1955-6-36

**Published:** 2014-09-11

**Authors:** Christina M Duzyj, Michael J Paidas, Lellean Jebailey, Jing Shun Huang, Eytan R Barnea

**Affiliations:** 1 Department of Obstetrics, Gynecology and Reproductive Sciences, Yale Women and Children’s Center for Blood Disorders, Yale University School of Medicine, 333 Cedar St, P.O. Box 208063, New Haven, CT 06520, USA; 2 GeneGo Inc., A Thomson Reuters Business, 5901 Priestly Drive Suite 200, Carlsbad, CA 92008, USA; 3 Department of Obstetrics and Gynecology, Reproductive Biology Unit, The Ohio State University, Columbus, OH 43210, USA; 4 Society for the Investigation of Early Pregnancy, 1697 Lark Lane, Cherry Hill, NJ 08003, USA; 5 BioIncept LLC (PIF Proprietary), 1697 Lark Lane, Cherry Hill, NJ 08003, USA

**Keywords:** Embryogenesis, Neural development, Preimplantation factor (PIF), Neural disease, Uterine environment

## Abstract

**Background:**

Intimate embryo-maternal interaction is paramount for pregnancy success post-implantation. The embryo follows a specific developmental timeline starting with neural system, dependent on endogenous and decidual factors. Beyond altered genetics/epigenetics, post-natal diseases may initiate at prenatal/neonatal, post-natal period, or through a continuum. Preimplantation factor (PIF) secreted by viable embryos promotes implantation and trophoblast invasion. Synthetic PIF reverses neuroinflammation in non-pregnant models. PIF targets embryo proteins that protect against oxidative stress and protein misfolding. We report of PIF’s embryotrophic role and potential to prevent developmental disorders by regulating uterine milieu at implantation and first trimester.

**Methods:**

PIF’s effect on human implantation (human endometrial stromal cells (HESC)) and first-trimester decidua cultures (FTDC) was examined, by global gene expression (Affymetrix), disease-biomarkers ranking (GeneGo), neuro-specific genes (Ingenuity) and proteins (mass-spectrometry). PIF co-cultured epidermal growth factor (EGF) in both HESC and FTDC (Affymetrix) was evaluated.

**Results:**

In HESC, PIF promotes neural differentiation and transmission genes (TLX2, EPHA10) while inhibiting retinoic acid receptor gene, which arrests growth. PIF promotes axon guidance and downregulates EGF-dependent neuroregulin signaling. In FTDC, PIF promotes bone morphogenetic protein pathway (SMAD1, 53-fold) and axonal guidance genes (EPH5) while inhibiting PPP2R2C, negative cell-growth regulator, involved in Alzheimer’s and amyotrophic lateral sclerosis. In HESC, PIF affects angiotensin via beta-arrestin, transforming growth factor-beta (TGF-β), notch, BMP, and wingless-int (WNT) signaling pathways that promote neurogenesis involved in childhood neurodevelopmental diseases—autism and also affected epithelial-mesenchymal transition involved in neuromuscular disorders. In FTDC, PIF upregulates neural development and hormone signaling, while downregulating genes protecting against xenobiotic response leading to connective tissue disorders.

In both HESC and FTDC, PIF affects neural development and transmission pathways. In HESC interactome, PIF promotes FUS gene, which controls genome integrity, while in FTDC, PIF upregulates STAT3 critical transcription signal. EGF abolished PIF’s effect on HESC, decreasing metalloproteinase and prolactin receptor genes, thereby interfering with decidualization, while in FTDC, EGF co-cultured with PIF reduced ZHX2, gene that regulates neural AFP secretion.

**Conclusions:**

PIF promotes decidual trophic genes and proteins to regulate neural development. By regulating the uterine milieu, PIF may decrease embryo vulnerability to post-natal neurodevelopmental disorders. Examination of PIF-based intervention strategies used during embryogenesis to improve pregnancy prognosis and reduce post-natal vulnerability is clearly in order.

## Background

A rapid and critical period of embryogenesis ensues post-embryo hatching and post-implantation; embryogenesis is then completed by 6 weeks post-implantation. During this period, the embryo mainly relies on itself for development, though this paradigm shifts radically towards placenta and mother later in gestation. The intimate contact established by the trophoblast with the maternal decidua enables effective exchange of nutrients and trophic agents. Since trophoblastic cells do not create a true barrier, the embryo benefits from the surrounding uterine milieu.

The earliest structure formed in the embryo post-implantation is the neural plate, with the spinal cord fold fusing by 6 weeks. The genes for proteins such as sonic hedgehog (SHH), wingless-int (WNTs), notch, bone morphogenetic proteins (BMPs), transforming growth factor beta (TGF-β), EPH, and others are implicated in cerebral cortical function and formation [1-3], as chick embryo and rat studies have revealed. Whether all of these regulatory factors are endogenous to embryonic cells, derived from the uterine milieu, or derived from a combination of both sources is not yet fully established. Moreover, it is important to determine whether the embryo itself (by regulating the maternal decidua) is an active participant in directing the flow of trophic compounds or is a passive participant in this process. Finally, embryo-derived secretory products acting on the uterine milieu may provide protection during gestation and possibly prevent disorders that can manifest long after birth. Evidence for such a protective effect derives from experimental observations that trophoblastic cells, and the embryo, effectively inactivate xenobiotics [4-9].

To understand the embryo’s role, examining embryo-specific compounds’ effect on the intrauterine milieu provides crucial information on embryo-induced conditioning involved in successful implantation and ensuing embryogenesis. Preimplantation factor (PIF) is a specific, post-fertilization peptide signal secreted by viable embryos. PIF is expressed both by the embryo/fetus and the placenta and is present in the maternal circulation throughout viable pregnancy [10-12]. PIF exerts four major complementary effects that are essential for pregnancy initiation and maintenance: 1) promoting the development of cultured embryos and acting as a rescue factor negating toxic-serum-induced embryo demise [13]; 2) promoting endometrial receptivity by regulating genes that are involved in inflammation, adhesion, and apoptosis and promoting the secretion of immune regulatory ligands and modulation of kinase phosphorylation, which actively condition the uterine environment [14,15]; 3) enhancing trophoblastic invasion, which provides effective oxygen and nutrient exchange for the fetus, an effect that is not synergized with epidermal growth factor (EGF) [16]; and 4) regulating systemic immunity to promote embryo tolerance while preserving the maternal ability to fight pathogens/disease and negating NK cell-induced toxicity [17,18].

As recently reported, PIF plays a determining role in the embryo’s neural development and neuroprotection by targeting the embryo proteins involved in oxidative stress, protein misfolding, and neural development. Specifically binding to protein-di-isomerase/thioredoxin (PDI/TRX) and heat shock proteins (HSPs) was noted. These major targets share a common binding site for PIF, enabling multi-targeting. In addition, PIF also targets tubulins, backbone of neurons [19]. PIF also upregulates decidual proteins that play a major role in neural function: agrin (a component of the neuromuscular junction), Calpain1 (a cytoskeleton component), NDUFS3 (modulator of oxidative stress), and PPF1BP1 (involved in axon guidance), in human implantation models [14].

PIF reverses advanced brain damage induced by hypoxia and inflammation in newborn rat models [20-22]. In chronic neuro-inflammation models, PIF reverses severe paralysis by reducing oxidative stress and protein misfolding while promoting and facilitating neural repair (by increasing neuron assembly and transmission) via local and systemic effects [23]. In a clinically relevant model for multiple sclerosis, subcutaneous administration of PIF led to reduced brain inflammation and reversal of paralysis. Notably, observations have demonstrated that PIF directly targets microglia, the major immune element within the CNS [24]. In this and other models, the primary PIF targets are insulin degrading enzyme (IDE) and potassium channel Kv1.3b, both of which play a prominent role in neural diseases and are targets for neuroprotective drugs [14].

The aim of the current study is to assess the impact and perform a more complete characterization of PIF’s effect on the maternal decidua, both at implantation and during the first trimester, in order to establish its embryotrophic and specifically neurotrophic roles in early gestation. For this, PIF’s impact on gene expression was examined in relation to 1) genes involved in trophic effects on the embryo with an emphasis on the neural system and 2) genes involved in potentially protecting against development of diseases in childhood and throughout adult life. In addition, 3) PIF’s specificity of action was examined by using EGF (a growth factor) as an antagonist. The data generated implies that the embryo, through PIF signaling, can condition the uterine milieu to regulate and mitigate the environmental impact on its own neural development. Thus, PIF, in addition to supporting embryo development, could play a role in decreasing the risk of developing post-natal disorders.

## Methods

### PIF peptide synthesis

The method was already described [25]. Briefly, synthetic PIF analog (MVRIKPGSANKPSDD) was produced using solid-phase peptide synthesis (Peptide Synthesizer, Applied Biosystems, Foster City, CA, USA) employing 9-fluorenylmethoxycarbonyl (Fmoc) chemistry. Final purification was carried out by reversed-phase high-pressure liquid chromatography, and peptide identity was verified by mass spectrometry as previously described [14].

### Endometrial cell cultures

Yale University School of Medicine review board approval was obtained for this study. Using our previously established cell culture method [14,15], non-pregnant human endometrial stromal cells (HESC) and cells collected from healthy first trimester of pregnancy deciduas were studied. Discarded endometrial tissue from premenopausal women undergoing hysterectomies due to benign indications was used. Decidual specimens from the first trimester were obtained from women undergoing elective termination in weeks 6–12 of normal pregnancy. Endometrial and decidual cells were isolated and re-suspended in Roswell Park Memorial Institute (RPMI)-1640 medium, grown to confluence, and found to be leukocyte free (<1%). After reaching confluence, the cells were decidualized using 10^-8^ mol/L estradiol and 10^-7^ mol/L synthetic progestin analog (R5020) (DuPont/NEN, Boston, MA, USA), in both cases for 7 days. The cells were switched to a serum-free medium containing insulin, transferrin, and selenium (Collaborative Research Inc., Waltham, MA, USA); 5 μmol/L trace elements (GIBCO, Carlsbad, CA, USA); and 50 μg/mL ascorbic acid (Sigma-Aldrich, St. Louis, MO, USA), and treated for 24 h with or without synthetic PIF (100 ng/mL). In other experiments, 50 ng EGF was cultured together with 100 ng/mL PIF for 24 h in HESC and first-trimester decidua cultures (FTDC). The tissue culture was collected and frozen at -80°C The samples were tested in triplicate.

### Microarray analysis

Total RNA was extracted from each cell culture. Analysis of HESC or FTDC with and without PIF 100 ng/mL (*n* = 3/group) or +/- EGF was examined using Affymetrix (Santa Clara, CA, USA) U133 Plus 2.0 Array (>38,500 human genes), followed by fluorescence labeling and hybridization with Fluidics Station 450 and optical scanning with GeneChip Scanner 3000 (Affymetrix) at W. M. Keck Foundation Biotechnology Resource Laboratory, Yale University, New Haven, CT, USA. Raw data were analyzed by GeneSpring software (Agilent, Santa Clara, CA, USA), normalized for inter-chip and intra-chip variations to eliminate false-positive results.

### Statistical analysis: gene pathway (MetaCore)

Genes that were significantly changed in expression by PIF (*p* < 0.05) using Student *t* test followed by a greater than twofold change were reported. Further results were divided into upregulated or downregulated lists and underwent new gene pathway analysis. MetaCore from GeneGo Inc., a Thomson Reuters business (Carlsbad, CA, USA), was used to identify and visualize the involvement of differentially expressed genes in specific cellular pathways. Enrichment analysis algorithms across several GeneGo ontologies were used to rank pathways, process, and diseases. To determine possible key regulators that may contribute to PIF-induced changes in gene expression to the human interactome (manually annotated interactions from peer-reviewed published experiments), the interactome tool in the MetaCore platform was used. To further understand the mechanisms and processes represented by the top connected hubs, networks were built using the shortest path algorithm followed by enrichment analysis using the disease and gene ontology processes.

### Statistical analysis of neural gene ranking (Ingenuity)

Pathway analysis was performed using the Ingenuity Systems Inc. (Redwood, CA, USA) software, ranking by greatest number of genes in a given pathway and associated statistical significance.

### Mass spectrometry analysis

HESC protein lysates (*n* = 3/group) from an independent experiment as compared to those of an mRNA study were homogenized in retinoic acid receptor alpha (RARA) buffer (100 mM Tris 250 mM NaCl, 0.1% Triton X-100, 0.1% sodium dodecyl sulfate (SDS)) and assayed for protein concentration. Twenty micrograms of each lysate was loaded in duplicate onto an SDS-poly-acrylamide gel Novex 4%–12% (Invitrogen, Carlsbad, CA, USA) followed by electrophoresis. Each lane was excised into 40 equal slices, digested with trypsin, and analyzed by nano-liquid chromatography/mass spectrometry (nano-LC/MS/MS) on an LTQ-Orbitrap XL™ tandem mass spectrometer (ThermoFisher, San Jose, CA, USA) carried out at NextGen Sciences (Ann Arbor, MI, USA). Data were searched against the Mascot concatenated forward-and-reversed v3.38 International Protein Index (IPI) database (Matrix Science Ltd., London, UK) and collated into non-redundant lists using Scaffold software (Proteome Software Inc., Portland, OR, USA). Using these combined forms of software creating ion identity score, and using the PeptideProphet provide high accuracy by determining true probabilities of proteins identified. Spectral counting was employed for relative quantitation, and a *t* test was utilized to show significant differences.

## Results

### PIF regulates neurodevelopmental gene expression at embryo implantation phase (HESC) and throughout the first trimester (FTDC)

Neural development initiates shortly post-implantation, and therefore, PIF’s effect on pathways that lead to the secretion of neurotrophic factors for the embryo by the decidua was examined (Table 1). The highest ranking gene (13.8-fold increase) is TLX2—a nuclear receptor subfamily 2 group E that binds DNA. This protein plays a major role in anterior brain differentiation and vision development [25]. Also, highly ranking is EPHA10 (9 fold) whose protein encodes a receptor for tyrosine kinase targeted by ephrin-A family members [26]. This protein is important for cell-to-cell communication and neural cell mobility. On the other hand, RARA gene expression decreased (-9 fold) [27]. This gene encodes a receptor for retinoic acid which is involved in growth arrest.

**Table 1:** Effect of PIF on HESC and FTDC genes involved in specific neural pathways

**HESC**		**FTDC**	
TGFβ			
TLX2	13.8	SMAD1	53.4
CREBBP	-2.2	SMAD6	2.7
SMAD6	-2.4	SMAD2	2.5
TGFBR1	-2.6	ACVR1C	-3.1
		INHBC	-4.6
EPH			
EPHA10	9.0	EPHA5	7.8
CDC42	3.7	RAP1A	2.3
EPHA5	2.4	EGF	2.2
ITSN1	-2.3	AKT3	2.2
PDGFC	-2.6	STAT3	-2.1
PTK2	-3.1	ANGPT1	-2.3
EPHA6	-3.1	RAC1	-2.4
AKT2	-5.6		
WNT			
MDM2	5.0	SOX17	2.5
LEF1	2.2	AKT3	2.2
CREBBP	-2.2	KREMEN1	-2.3
TLE4	-2.3	CSNK2A1	-2.3
CDH2	-2.5	PPP2R1B	-2.5
TGFBR1	-2.6	ACVR1C	-3.1
WNT16	-3.9	PPP2R2C	-6.6
AKT2	-5.6		
RARA	-9.4		

In FTDC, the major upregulated gene was SMAD1 (53.4-fold increase) (Table 1) [28]. Its encoded protein is TGFβ signaling protein, a major modulator of the bone morphogenetic protein—highly relevant for embryo neural development [29]. There is also an increase in SMAD6 and SMAD2 (2.7-fold and 2.5-fold, respectively). EPHA5 increased by 7.8-fold; this protein is a receptor tyrosine kinase that binds ephrin-A family ligands, which are highly relevant to neural development, especially axonal guidance and synaptogenesis [30]. On the other hand, PIF downregulates PPP2R2C, which is involved in negative control of cell growth and division [31]. Collectively, this set of data indicates that PIF is involved in pathways that are critical for neural development.

### PIF’s neurotrophic effects: Ingenuity analysis of neural-related pathways in HESC and FTDC

PIF also appears to affect several neural-related pathways that could contribute to neurotrophic effects on the embryo. The highest correlation was found with axon guidance signaling followed by neuroregulin, which is involved in EGF signaling; the specific genes involved are listed in Table 2. Effective synaptic transmission is recognized to be critical for effective neural development. The regulatory effect of PIF in this pathway reflects the peptide’s significant protective role against altered neural development. The lowest ranking—but still relevant—gene pathway affected by PIF is the oxidative phosphorylation pathway, which is also critical for embryo survival.

**Table 2:** PIF ranking and effect on neurotrophic gene expression in HESC

**Axonal guidance signaling 9.20E - 02**		**Neuregulin signaling 8.70E - 02**		**Synaptic long-term potentiation 7.46E - 02**		**Amyotrophic lateral sclerosis signaling 6.94E - 02**		**Synaptic long-term depression 4.84E - 02**		**Huntington’s disease signaling 4.90E - 02**		**Oxidative phosphorylation 3.47E - 02**	
CDC42	3.7	PTEN	2.6	CAMK2B	2	RAB5A	3.9	PRKG1	-2.5	CREBBP	-2.1	ATP6V0D2	3.5
EPHA5	2.3	ERBB2IP	-2.2	CAMK2D	-2.1	GRIK5	2.3	PRKCB1	-4.4	PIK3CA	-2.4	PPA2	3.5
SEMA4C	2	PDK1	-3	CREBBP	-2.1	GRIK2	-2	GRM1	-17	HDAC9	-3.7	CYB5A	3.1
PDGFC	-2.5	ERBB3	-3.2	PRKCB1	-4.4	PIK3CA	-2.4			CASP8	-4.2	NDUFA10	2.1
SEMA6A	-4.5	PRKCB1	-4.4	GRM1	-17	BCL2	-21			PRKCB1	-4.4	NDUFA5	-2.1
SEMA4D	-6	AKT2	-5.6							AKT2	-5.6		
SEMA3D	-10									GRM1	-17		
SEMA3C	-11												

Upon analyzing the FTDC data (Table 3), an interesting pattern emerged since the data showed that the highest ranking neural pathways affected by PIF are actually related to adult neurological disorders. Among them are amyloid processing genes that can lead to Alzheimer’s disease, amyotrophic lateral sclerosis, and Huntington’s chorea. This is followed by effects on neural signaling pathways. PIF’s effect on individual genes and their expression in the different pathways is shown in Table 3. This correlation with adult disease implies an evolution in PIF targeting of the maternal milieu with advancing gestation which is different from earlier development phases which appear to be more involved in early childhood diseases.

**Table 3:** PIF ranking and effect on neurotrophic genes expression in FTDC

**Amyloid processing 8.33E - 02**		**Amyotrophic lateral sclerosis signaling 8.33E - 02**		**Huntington’s disease signaling 7.69E - 02**		**Synaptic long-term depression 6.45E - 02**		**Axonal guidance signaling 5.75E - 02**		**Neurotrophin/TRK signaling 4.00E - 02**		**Neuregulin signaling 1.16E - 01**	
AKT3	2.1	GRIK2	2.5	BDNF	3	GNA12	-2	ARHGEF12	9	BDNF	3	NRG1	5
CDK5R1	2	RAC1	-2.4	AKT3	2.1	PPP2R1B	-2.5	EPHA5	7.8	CSNK2A1	-2.3	PLCG2	3.1
CSNK2A1	-2.3	SLC1A2	-3	EGF	2.1	GUCY1A3	-4.9	GLI2	6.1			EGF	2.1
		PPP3CA	-9.2	POLR2J2	2	PPP2R2C	-6.5	NRP1	4.2			AKT3	2.1
		BCL2	-27	CDK5R1	2			BDNF	3			CDK5R1	2
				TCERG1	-2			RAP1A	2.2			ADAM17	-2
				SNCA	-2			AKT3	2.1			PDK1	-5.2
				CLTC	3.1			EGF	2.1			EGFR	-24.5
				RPH3A	-3.1			DOCK1	2.1				
				EGFR	-24.5			SEMA6D	2.1				
								SLIT1	2.1				
								RAC1	-2.4				
								ROBO1	-2.5				
								DPYSL5	-4.6				
								PPP3CA	-9.2				

### PIF’s effects on HESC and FTDC—pathways and protection against post-natal disease—MetaCore analysis

Since PIF exhibits trophic effects mainly on neural development, it was of interest to further examine the genes involved in embryo development as well, possibly a long-term protective strategy to prevent adverse post-natal disorders. The MetaCore program enabled such analysis. The full description of the role of PIF in multiple cellular signaling pathways is however beyond the scope of this manuscript. Hence, this information is available in detail in Additional file 1 (pages 1–42), and only key findings are referenced herein. For such a comprehensive analysis, the data had to be analyzed as pathway maps, process networks, followed by the specific physiologic processes involved, and finally the disease association, when these processes are perturbed which lead to disease during childhood or in adulthood (Table 4). Data was analyzed as the highest association in each category, with associated statistical significance.

**Table 4:** PIF effect on HESC analyzing maps, processes, disease ranking, and strength of association

**GeneGo pathway maps**		**GeneGo process networks**		**GO processes**		**GeneGo diseases (by biomarker)**	
**Name**	***p *** **value**	**Name**	***p *** **value**	**Name**	***p *** **value**	**Name**	***p *** **value**
Development_angiotensin signaling via beta-arrestin	0.001218	Neurophysiological process_transmission of nerve impulse_	1.114E **-** 06	Nervous system development	1.736E **-** 16	Autistic disorder	4.026E **-** 14
Development_regulation of epithelial-to-mesenchymal transition (EMT)	0.001379	Muscle contraction	0.0005106	Positive regulation of biological process	7.282E **-** 16	Child development disorders, pervasive	4.887E **-** 14
G-protein signaling_RhoB regulation pathway	0.003256	Cytoskeleton_regulation of cytoskeleton rearrangement	0.0009131	Cell communication	5.021E **-** 15	Mood disorders	2.655E **-** 09
Cell adhesion_tight junctions	0.004814	Development_neurogenesis: axonal guidance	0.001197	Regulation of transport	9.227E **-** 15	Stress	2.787E **-** 09
Muscle contraction_GPCRs in the regulation of smooth muscle tone	0.005149	Reproduction_GnRH signaling pathway	0.008079	Anatomical structure development	1.207E **-** 14	Mental disorders diagnosed in childhood	6.579E **-** 09
Cytoskeleton remodeling_TGF, WNT, and cytoskeletal remodeling	0.005462	Cell adhesion_cell junctions	0.01565	Regulation of localization	1.48E **-** 14	Aortic diseases	6.6E **-** 09
Atherosclerosis_role of ZNF202 in regulation of expression of genes involved in atherosclerosis	0.007208	Signal transduction_cholecystokinin signaling	0.02107	Regulation of amine transport	1.744E **-** 14	Aortic aneurysm	6.801E **-** 09
Transcription_CREB pathway	0.009855	Signal transduction_TGF-beta, GDF, and activin signaling	0.02518	Transmission of nerve impulse	2.15E **-** 14	Craniomandibular disorders	1.025E **-** 08
Cell adhesion_role of CDK5 in cell adhesion	0.01212	Signal transduction_WNT signaling	0.02811	Positive regulation of cellular process	2.762E **-** 14	Temporomandibular joint disorders	1.025E **-** 08
Development_TGF-beta-dependent induction of EMT via MAPK	0.01239	Reproduction_gonadotropin regulation	0.02941	Regulation of multicellular organismal process	3.763E **-** 14	Mandibular diseases	5.041E **-** 08

PIF’s highest ranking pathway in HESC is the angiotensin signaling via beta-arrestin, which is coupled with neural transmission or nerve impulse associated with nervous system development and, when affected, leads to autism. The second ranking pathway affected by PIF was genes involved in the epithelial-mesenchymal transition (EMT), which is associated with muscle contraction, is needed for neural functionality, and if affected, can lead to child development disorders. Interestingly, PIF was found to have significant associations with pathways relating to mood disorders and stress, TGFβ, WNT, CDK5, and BMP.

Among the significantly upregulated processes for which PIF increased the gene expression more than twofold are (Table 5) angiotensin—via beta arrestin, G-protein signaling RhoA/B, and CDC42, showing that the effects on neurogenesis and immune response are dominant. In contrast, the processes downregulated by PIF are EMT, EGF activation, and axon growth repulsion. The balance among upregulated and downregulated processes implies a dynamic adaptive effect on these pathways.

**Table 5:** Summary of enrichment analysis for HESC and FTDC (genes that are expressed more than twofold or decreased twofold) regulating processes following PIF treatment

	**HESC**		**FTDC**	
**Ontology**	**Upregulated processes**	**Downregulated processes**	**Upregulated processes**	**Downregulated processes**
GeneGo Pathway Maps	G-protein signaling_RhoB regulation pathway	Development_TGF-beta-dependent induction of EMT via MAPK	Cell cycle_role of 14-3-3 proteins in cell cycle regulation	Androstenedione and testosterone biosynthesis and metabolism p.2
	Development_angiotensin signaling via beta-Arrestin	Immune response_CD137 signaling in immune cell	Development_EGFR signaling via PIP3	Acetaminophen metabolism
	G-protein signaling_RhoA regulation pathway	Development_regulation of epithelial-to-mesenchymal transition (EMT)	Apoptosis and survival_role of CDK5 in neuronal death and survival	1-naphthylamine and 1-nitronaphthalene metabolism
	Immune response_antiviral actions of Interferons	Cell adhesion_role of tetraspanins in the integrin-mediated cell adhesion	Development_neurotrophin family signaling	Immune response_IL-15 signaling via JAK-STAT cascade
	Immune response_IFN alpha/beta signaling pathway	Development_beta-adrenergic receptors transactivation of EGFR	*Neurophysiological process_receptor-mediated axon growth repulsion*	2-naphthylamine and 2-nitronaphthalene metabolism
	Development_signaling of beta-adrenergic receptors via beta-arrestins	Development_gastrin in differentiation of the gastric mucosa	Role of alpha-6/beta-4 integrins in carcinoma progression	Estradiol metabolism
	G-protein signaling_regulation of CDC42 activity	*Neurophysiological process_receptor-mediated axon growth repulsion*	Cell cycle_cell cycle (generic schema)	*G-protein signaling_regulation of RAC1 activity*
	Muscle contraction_GPCRs in the regulation of smooth muscle tone	Transcription_androgen receptor nuclear signaling	Development_ERK5 in cell proliferation and neuronal survival	Immune response_IL-15 signaling via JAK-STAT cascade
	Neurophysiological process_EphB receptors in dendritic spine morphogenesis and synaptogenesis	Regulation of metabolism_triiodothyronine and thyroxine signaling	Cell cycle_initiation of mitosis	Benzo [a] pyrene metabolism
	*G-protein signaling_regulation of RAC1 activity*	Cell adhesion_integrin-mediated cell adhesion and migration	G-protein signaling_K-RAS regulation pathway	Apoptosis and survival_HTR1A signaling

Listed are the top ten results from the Analyze Single Experiment Workflow across two ontologies. This table shows the upregulated and downregulated processes where the same processes between the two time points are italicized.

In FTDC (Table 6), on the other hand, different maps to diseases were identified. PIF’s highest ranking pathway was acetaminophen metabolism (i.e., xenobiotics) which relates to reproduction/hormone signaling and, when affected, can lead to autoimmune connective tissue disorders (Table 5). The second ranking pathway was steroid metabolism related to vasculogenesis and, when altered, leads to development of skin disorders. Of note, connective tissue disorders and neoplasms occur mostly in adults. The associated upregulated processes are cell cycle role in 14-3-3, ERK5, and mitosis, and neuronal survival and development. The downregulated processes are xenobiotics related to acetaminophen, 2-napthalamine, benzo (a) pyrene, and steroid metabolism. The emphasis on protection against xenobiotics is critical for PIF’s protective role during embryogenesis.

**Table 6:** PIF effect on FTDC analyzing maps, processes, disease ranking, and strength of association

**GeneGo pathway maps**		**GeneGo process networks**		**GO processes**		**GeneGo diseases (by biomarker)**	
**Name**	***p *** **value**	**Name**	***p *** **value**	**Name**	***p *** **value**	**Name**	***p *** **value**
Acetaminophen metabolism	0.00001014	Reproduction_feeding and neurohormones signaling	0.0004425	Multicellular organismal process	2.563E **-** 14	Skin and connective tissue diseases	1.006E **-** 10
Androstenedione and testosterone biosynthesis and metabolism p.2	0.00003172	Development_blood vessel morphogenesis	0.003215	Regulation of biological quality	9.359E **-** 14	Skin diseases	1.014E **-** 09
Cell cycle_role of 14-3-3 proteins in cell cycle regulation	0.00003564	Muscle contraction	0.003752	Positive regulation of biological process	1.611E **-** 13	Neoplasms by histologic type	2.396E **-** 09
Androstenedione and testosterone biosynthesis and metabolism p.2/rodent version	0.0000375	Cell cycle_meiosis	0.00434	Regulation of multicellular organismal process	3.408E **-** 13	Myoepithelioma	1.598E **-** 08
Development_EGFR signaling via PIP3	0.00004489	Cell cycle_G2-M	0.005763	Regulation of localization	4.975E **-** 13	Neoplasms, glandular, and epithelial	2.089E **-** 08
Cell cycle_initiation of mitosis	0.00006886	Development_ossification and bone remodeling	0.007848	Regulation of secretion	8.326E **-** 13	Capillary leak syndrome	6.196E **-** 08
Development_prolactin receptor signaling	0.00007228	DNA damage_DBS repair	0.00865	Intracellular signaling cascade	1.943E - 12	Breast neoplasms	8.058E **-** 08
Apoptosis and survival_role of CDK5 in neuronal death and survival	0.0003167	Development_regulation of angiogenesis	0.01318	Cellular calcium ion homeostasis	2.698E **-** 12	Breast diseases	8.287E **-** 08
Estradiol metabolism	0.0003642	Reproduction_male sex differentiation	0.01432	Calcium ion homeostasis	4.215E **-** 12	Schizophrenia	1.035E **-** 07
G-protein signaling_regulation of RAC1 activity	0.0004169	Translation_regulation of initiation	0.0149	Cellular metal ion homeostasis	4.501E **-** 12	Connective tissue diseases	1.222E **-** 07

### Similarity between PIF’s effect on HESC and FTDC

To document the dynamic gestational age-dependent effect of PIF in early pregnancy, similarities and differences between those critical time periods were examined. Common pathways affected by PIF include muscle contraction and angiotensin signaling via four independent pathways: STATs, ERK, PYK2, and CREB pathways. Neurophysiologic processes affected were melatonin signaling and receptor-mediated growth axon repulsion. Global processes were transmission of nerve impulses, axonal guidance, and cell adhesion. The similarity between the two time periods with respect to neural development illustrates PIF’s continuing role in this critical process.

### Interactome analysis of genes affected by PIF

To better delineate a network of genes associated with PIF activity, an interactome analysis was carried out. This enables us to relate a large number of genes that are linked in a network. The hub enables to define the number of interactions among genes. Table 7 illustrates the leading genes and their interaction. In HESC, FUS was dominant [32]. This gene controls genome integrity and RNA processing and has the highest (*n* = 68) interactions, followed by HIPK2, whose protein is a kinase involved in p53-regulated cellular apoptosis [33].

**Table 7:** Interactome analysis of PIF-induced hubs of genes more and less than twofold in both HESC and FTDC as it is related to protein function analysis

**HESC**			**FTDC**		
**HUB**	**Number of interactions with interactome (total hubs = 21,579)**	**Number of interaction with PIF-HESC genes (total hubs = 363)**	**HUB**	**Number of interactions with interactome (total hubs = 21,579)**	**Number of interaction with PIF-FTDC genes (total hubs = 384)**
ATBF1	19	3	PEA3	239	12
NK31	37	4	STAT3	603	26
Plexin A4	12	3	c-Rel	382	16
HIPK2	60	7	CDK1	389	22
SYNJ2BP	37	5	UGT1A6	9	3
FUS	68	7	UGT1A1	31	5
			Beta-fodrin	65	8

In FTDC, the number of interactions was much higher.

The FTDC interactions were much more extensive (Table 7). The highest ranking was STAT3 (*n* = 603) [34]. The protein plays a central role in JAK/STAT signaling of several cytokines. The next ranking was CDK1 (*n* = 389), a kinase that controls cell cycle regulation [35]. Of note, the decidua genes are more connected through two signaling steps than the genes found in HESC, in which three steps are involved.

Table 8 illustrates the top biological processes affected by PIF in HESC. The highly prominent DNA-dependent regulation is required during rapid cellular development. In contrast, the top process in FTDC is the response to chemical stimulus, i.e., protection against adverse environment during embryogenesis. This further substantiated the evolving role of PIF from supporting neural development to protection against xenobiotics.

**Table 8:** Top biological process of Interactome Hub networks, associated with statistical significance and major genes involved in the process

**HESC**				**FTDC**			
**Process**	**Percentage**	***p *** **value**	**Expressed genes**	**Process**	**Percentage**	***p *** **value**	**Expressed genes**
Regulation of transcription, DNA-dependent	74.07	1.216E - 28	NKX3-1, SP3, SOX2, STAT3, ZFHX3	Response to chemical stimulus	69.84	4.365E - 24	
Regulation of RNA metabolic process	74.07	3.141E - 28		Response to organic substance	57.14	4.814E - 24	UGT1A1, STAT3
Regulation of transcription from RNA polymerase II promoter	59.26	4.227E - 28	STAT3	Positive regulation of biological process	71.43	5.785E - 24	
Positive regulation of gene expression	57.41	6.061E - 28	MDM2	Positive regulation of macromolecule metabolic process	50.79	2.78E - 21	
Positive regulation of transcription	55.56	4.355E - 27	NKX3-1, SOX2, STAT3	Organ development	63.49	3.189E - 21	
Transcription	74.07	1.364E - 26	NKX3-1, SP3, SOX2, STAT3, HIPK2, ZFHX3	Positive regulation of cellular process	65.08	3.603E - 21	
Regulation of transcription	79.63	2.039E - 26	NKX3-1, SP3, SOX2, STAT3, HIPK2, ZFHX3	Positive regulation of cellular metabolic process	50.79	1.333E - 20	
Positive regulation of nucleobase, nucleoside, nucleotide, and nucleic acid metabolic process	55.56	1.925E - 25		Positive regulation of gene expression	42.86	4.074E - 20	CDK1
Positive regulation of transcription, DNA-dependent	50	3.096E - 25	HIPK2	Positive regulation of metabolic process	50.79	5.766E - 20	
Positive regulation of RNA metabolic process	50	3.955E - 25		Response to stimulus	77.78	4.007E - 19	
Positive regulation of macromolecule biosynthetic process	55.56	4.246E - 25		System development	66.67	4.06E - 19	
Positive regulation of nitrogen compound metabolic process	55.56	5.851E - 25		Positive regulation of transcription, DNA-dependent	38.1	4.184E - 19	TP63, REL

### PIF affects expression of HESC proteins

Using an independent experiment, the effect of PIF on a number of protein levels was determined by using semi-quantitative mass spectrometry (14). To provide a more detailed analysis, we have analyzed, in addition, the effect of PIF on a total of >1,300 HESC proteins demonstrating that several of them were affected by PIF (Additional file 2). We found that PIF affected several proteins involved in the control of oxidative stress and protection against abnormal protein synthesis: PDIA3, P4HB (PDI), TXND, PRD6, HSPB1, SDHA, and BAG1. This was clearly in line with the microarray data. In addition, specifically focusing on proteins involved in neural function, the data revealed that PIF affects several additional proteins among them. PPIC is affected in ataxia and in degeneration of Purkinje cells [36]. OCRL is affected in Lowe syndrome oculo-cerebral defects [37]. AP3D1 is involved in budding vesicle transmission to neurons [38]. SLC25A1 and SLC16A1 are proteins involved in mitochondrial transport widely expressed in the central nervous system [39]. LDHA is involved in substania nigra development and is deregulated in glioma tumors [40]. Thus, PIF also affects not only genes but also decidual proteins.

### Reciprocal negative interaction between PIF and EGF in HESC and FTDC

**Figure 1:**
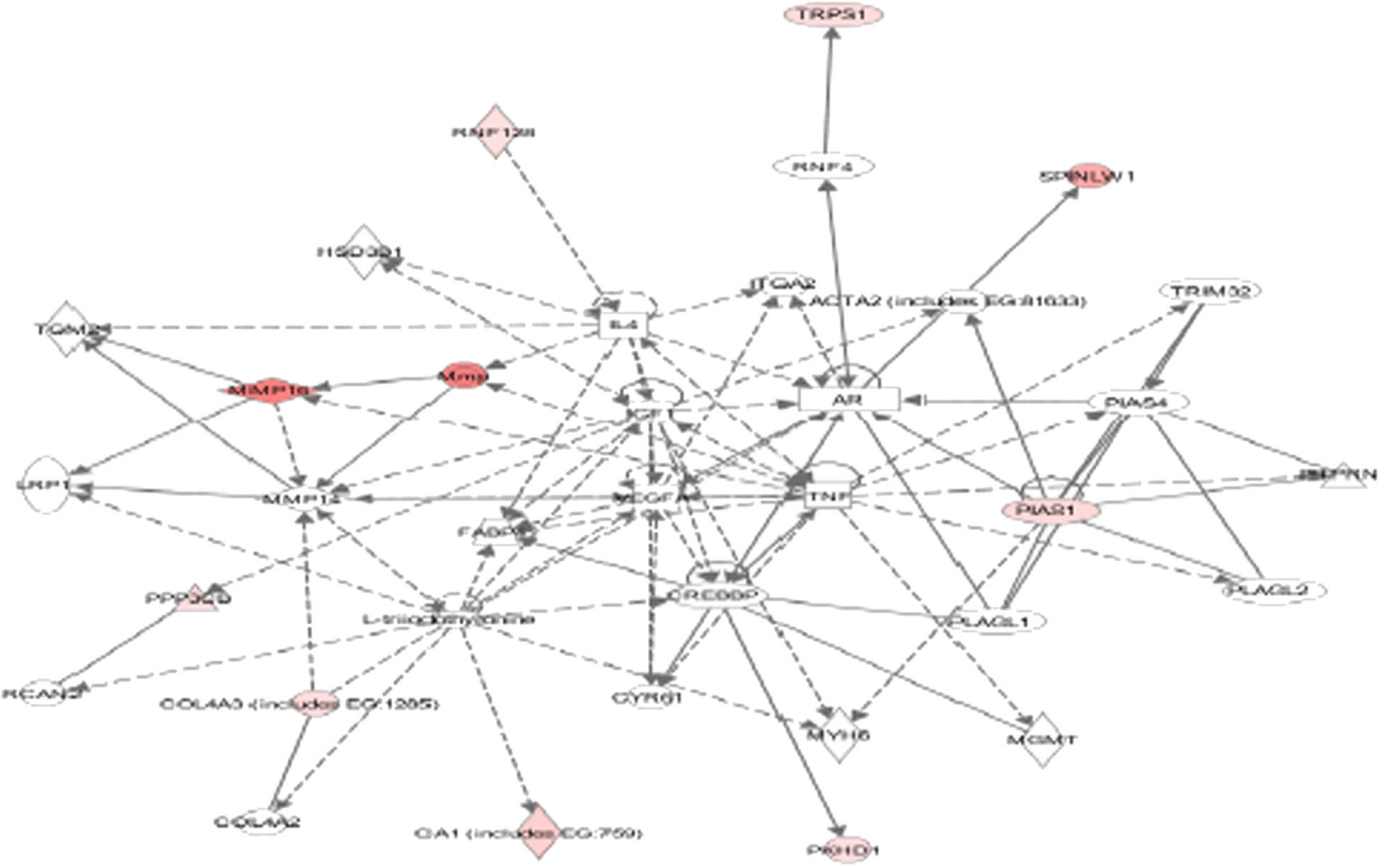
Pathway analysis as it relates to EGF signaling in HESC.

**Figure 2:**
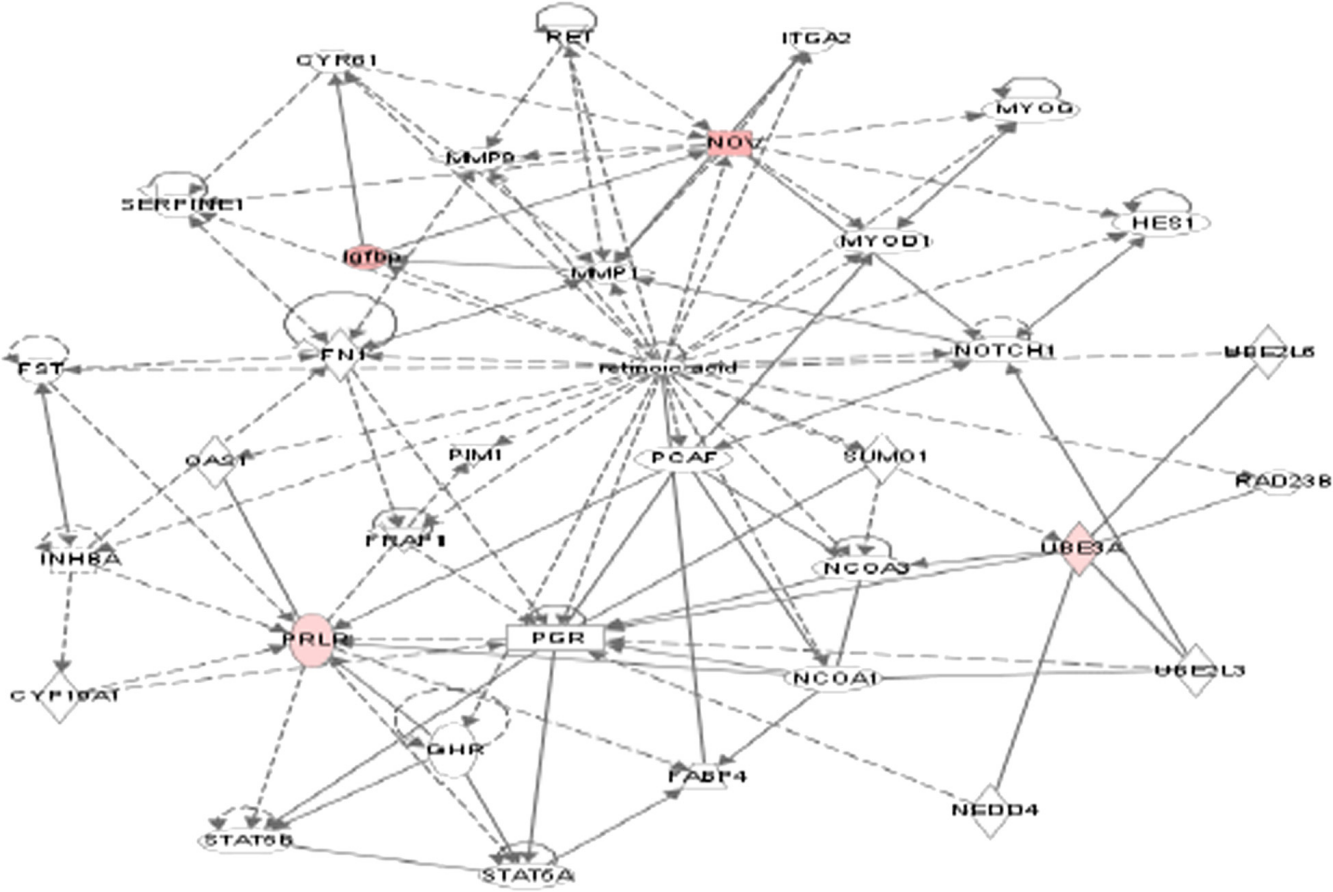
Pathway analysis as it relates to prolactin receptor gene in HESC.

EGF is reported to block decidua formation, and PIF was shown to regulate EGF-related pathways, promoting amphiregulin and epiregulin while inhibiting betacellulin [15]. Therefore, whether EGF can affect PIF’s action was examined. Addition of EGF abolished PIF’s effect on the global genome. Only a limited number of genes (*N* = 15) were affected, mostly of which were downregulated. The EGF treatment-related pathway is shown (Figure 1). The decrease noted in MMP metalloproteinase expression may interfere with decidual function [41]. This is further amplified by the decrease in prolactin receptor (Figure 2), where the ligand is crucial for decidua formation. The most downregulated gene was BMPER, an inhibitor of BMP (-10 fold) followed by NIF3LBP1 (-7.4 fold), which binds NIF3 (an amyotrophic lateral sclerosis candidate gene), MCC1 (-7.4 fold) (a tumor suppressor), and PRLR (-4.2) (a prolactin receptor important for decidua function) [42-46]. The gene polymorphism is associated with multiple sclerosis. The only gene which was increased is Nov (2 fold), which is a negative regulator of cell growth in choriocarcinoma cells [47].

**Figure 3:**
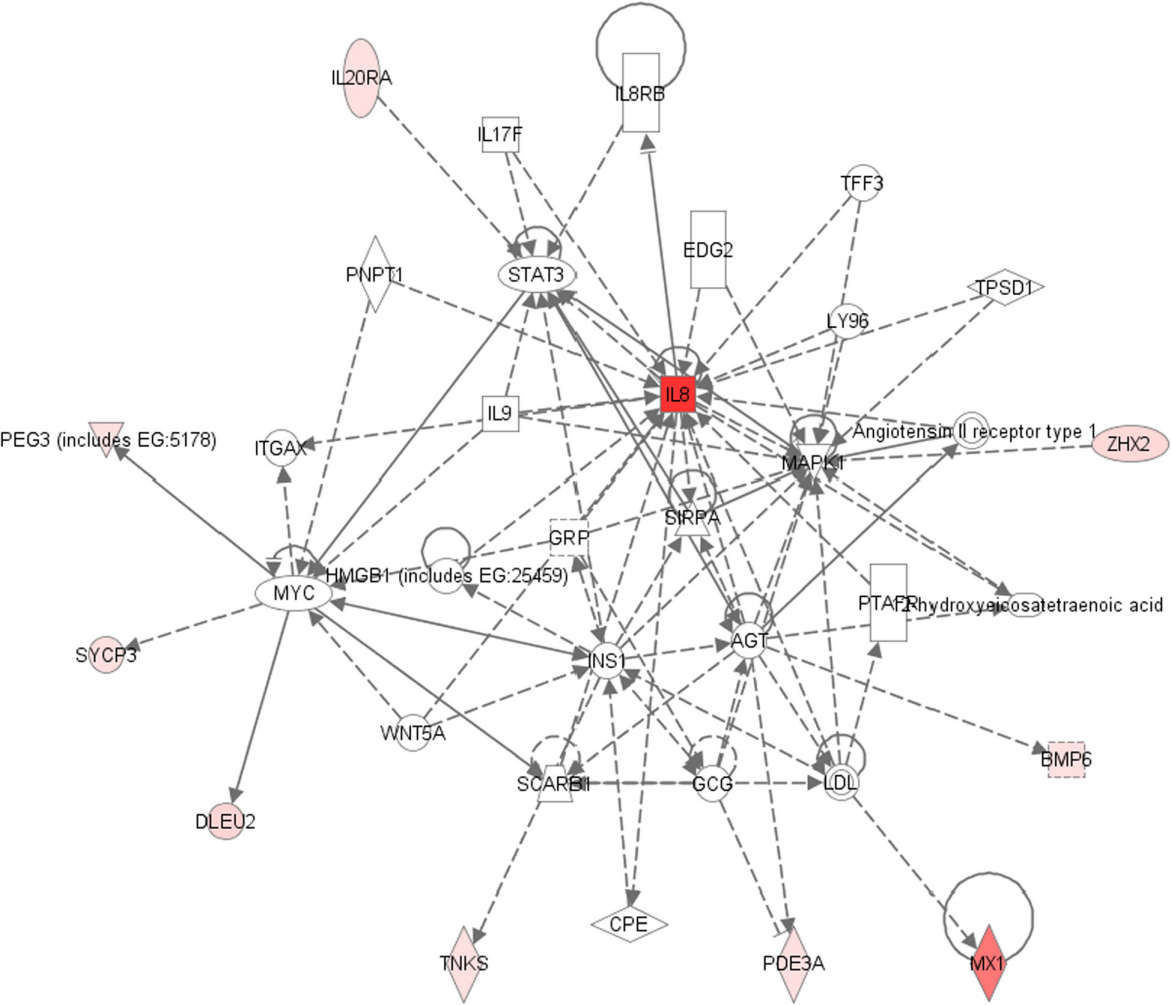
Pathway analysis as it relates to PEG3 gene in FTDC.

PIF significantly reduces EGF receptor (-25.6 fold) in FTDC, therefore blocks the ligand’s activity [14]. Whether EGF interferes with PIF’s action on the decidua was analyzed. The most downregulated gene was ZHX2 (-8.4 fold), which was shown to promote AFP secretion leading to activation in liver cancer [48]. Figure 3 shows that EGF reduced IL8 expression and decreased PDE3A (-5.6 fold), involved in learning and memory [49]. There was also a decrease in PEG3, which blocks inflammation-induced apoptosis and is a tumor suppressor in glioma cells (-6.3 fold). Interestingly, PIF alone on FTDC negated both PEG3 (-5 fold) and PDE7B (-5.7 fold) [50,51]. These observations document the negative relationship between PIF, a promoter of decidual function, and EGF, which acts as a pro-proliferative agent with anti-decidual properties.

## Discussion

After hatching but before being protected by the placenta, the embryo is highly vulnerable to maternal adversity. However, the embryo has developed efficient protective mechanisms. It is herein demonstrated that PIF, an embryo-secreted peptide, differentially conditions the maternal uterine environment during implantation and the first trimester. By regulating the uterine milieu, PIF provides crucial embryotrophic support for the neural system, which is of central importance to the early embryo’s development and has deep consequences throughout early as well as throughout adult life. During implantation, PIF’s major effect is exerted on neurotrophic genes that are involved in regulating signaling involved in neurodevelopmental disorders. PIF, in the first trimester decidua during embryogenesis, affects signaling pathways that are involved in protecting against xenobiotic exposure and regulates pathways involved adult neurodegenerative and autoimmune connective tissue disorders. In contrast, EGF, a potent growth factor, is a PIF antagonist and abolishes its protective effects on the decidua. This demonstrates that PIF’s embryotrophic and protective effect is dynamic and dependent on the conditioning of the maternal milieu.

Numerous decidual genes related to neural function and development affected by exposure to PIF are herein characterized and analyzed. This complements and expands observations of PIF’s effect on genes associated with secreted products and phosphorylated kinases that can aid in embryo development [14,15]. The observation that post-implantation PIF is present in the maternal circulation and is expressed by the placenta creates an intimate link between this embryo/trophoblast-secreted peptide and the maternal uterine environment [11]. The use of two advanced modes of gene analysis (Ingenuity and MetaCore) reveals complex mechanisms involved in PIF’s action.

PIF’s gene/pathway regulation was examined as it is related to embryotrophic effects. Post-implantation, the neural plate forms via migration of primary cells, with fusion of the neural folds within 30 days post-conception; the upper segment then forms the brain, while the lower segment forms the spinal cord. Several trophic factors are involved in neural formation, including the TGFβ, BMP-2, EPH-1, and WNT-3 pathways [1-3]. Some trophic factors are endogenous to the embryo (which has a very small surface area for synthesis/secretion), while other trophic factors are of decidual origin which has a larger surface area and are likely to be dominant.

PIF upregulates decidual trophic genes, several of which promote neural system development. Specifically, during the implantation period, increases are noted in TLX2 and EPHA10 (a brain trophic agent and an agent that improves neural transmission, respectively). Conversely, PIF lowers RARA expression, preventing growth arrest. This was further emphasized in PIF’s neural pathway ranking where the top ranking was in genes promoting axonal guidance and synaptic potentiation. Remarkably, in FTDC, in which the embryo/maternal relationship has already been well established, PIF significantly promotes SMAD1, which is involved in the BMP pathway and is critical for embryo neural development. With regard to pathway ranking in FTDC, PIF’s more prominent effects are on genes relating to post-natal diseases since the embryo’s neural fold has already been closed at this stage.

The MetaCore program provides a unique insight into gene regulation, pathways, and association with possible diseases. Analyzing in detail PIF’s effects on disease-related pathways in the two different gestational times identifies a complex picture, since the number of genes affected is very large. This indicates that PIF’s effect is robust, especially with respect to the angiotensins (mainly angiotensin II), which serve as brain modulatory factors implicated in stress response and vulnerability to cerebrovascular ischemia and inflammation. Angiotensin receptor blockers may protect neurons [52]. Angiotensin IV and its receptor are implicated in cognitive processing and memory impairment [53]. Specific downstream pathways via CREB and Akt are neural progenitor regulators [54] and targeted by using rapamycin against neonatal hypoxia and ischemia [55]. Additionally, the angiotensin family (1–7) increases neuronal voltage-gated potassium current through nitric oxide pathway [14,56]. Furthermore, viral infections cause, in the prenatal period, activation of the inOS pathway in murine models [57]. We demonstrated that PIF controls the inflammatory response in macrophages by blocking this specific pathway [58]. Therefore, the novel association found between PIF and angiotensin warrants further examination in targeting neurological diseases.

PIF also had a significant effect on genes involved in the EMT [59]. This implies that PIF can affect cells that are involved in enabling transformation of one cell type to another, reversibly critical for proper embryo development. On the other hand, under inflammatory conditions, EMT can also be effectively activated, harming the host. The major factors involved are BMP7 and Wilms tumor 1 genes. As a consequence of this regulation, PIF affects genes involved in post-natal neurologic diseases such as autism and later onset neurodegenerative diseases like multiple sclerosis. Thus, altered embryo-maternal communication may increase the susceptibility for such disorders.

Endometrial inflammation is a frequent pathologic environment. When subtle, there may be no clear embryo rejection leading to spontaneous abortion; however, there may still be long-term adverse consequences that can manifest post-partum. PIF may play a role in averting such adverse events. As expected in the decidua, PIF’s effect on xenobiotic metabolism and hormonal control are prominent; however, the more subtle effect on neurodevelopmental diseases is still evident, mostly their expression in adult diseases.

Our interactome analysis provided further insight into PIF’s gene network, identifying those specifically involved. Interestingly, the linkage between genes was more tightly regulated in the FTDC. Perhaps this is related to the fact that the decidual cells were primary, as compared to HESC, which were steroid-activated stromal cells. Overall, the reciprocal relationship between the embryo through PIF and the maternal decidua appears to play an important role in the earliest stages of development. As the genes affected at implantation and FTDC are different, this is a further confirmation of PIF’s selective and targeted effect on the maternal environment.

Further substantiating observations made on PIF regulatory effects on HESC proteins (14), we have examined PIF’s effect on the global proteome. PIF affects proteins PDI and HSPs that reduce oxidative stress and protein misfolding. Remarkably, PDI and HSP are also PIF-binding targets (19). Therefore, we further document that PIF not only regulates HESC but also identifies through which proteins such a targeting most likely such effect takes place. Therefore, such data creates an intimate integration between effect and site of action.

The highly ranking neuroregulin pathway in HESC is EGF related. PIF inhibits this pathway by downregulating ERB03, ERBB2IP, and PRKCB-, pro-proliferative genes further amplified by the increase in PTEN, a tumor suppressor. Conversely, EGF completely blocked PIF’s effect on both HESC and FTDC, confirming the antagonistic effect of the growth factor. This is expected since EGF promotes decidual cell proliferation and has anti-decidual effects [60]. It is of note that decidual heparin-binding epidermal growth factor-like growth factor (HB-EGF) has distinct properties as compared with EGF [61]. Such inhibitory action indicated also that PIF’s effect is dynamic and is dependent on a given decidual environment.

From a clinical perspective, genetic defects and epigenetic modifications are major contributors to post-natal disease. Development of invasive and non-invasive diagnostic tests has led to a significant decrease in the birth of affected children. Despite these advances, *in utero* identification of more subtle neurodevelopmental disorders is still lacking. In autism, a condition whose incidence continues to rise, recent reports indicate that two complementary brain pathologies co-exist; immune over-activity and reduced neural interaction [62]. Thus, the syndrome may initiate in the prenatal period, as supported by murine models [63]. Consequently, it is important to address how early such defects initiate and whether they are preventable or treatable if identified early. Neural tube defects, and possibly other neurodevelopmental disorders, may also develop during early embryogenesis. Evidently, major neurologic disorders such as anencephaly will be self-limiting or diagnosed and eliminated, whereas more subtle forms might persist. Insight into these processes could move forward the field of neurodevelopment, with significant implications for post-natal life.

## Conclusions

The current study supports the view that the embryo, through PIF, conditions the uterine environment to reduce the impact of adverse maternal environment. In addition to structural imaging, several serum markers (hCG, PAPP-A, estriol, and AFP) can identify neurodevelopmental disorders such as Down’s syndrome and neural damage. Recently, metabolites related to oxidative stress were found to be increased in the serum of patients with Down’s syndrome [64]. These observations imply that maternal conditioning may play a significant role in regulating embryonic neural development. It is suggested here that PIF could improve such maternal conditioning.

The study is limited since PIF’s effects were assessed in culture and therefore cannot directly reflect the *in vivo* environment. However, the two time-point models used are well established to provide important insight into the biology of early pregnancy. A further strength is the use of two complementary, advanced methods of data analysis which independently confirm the observation that PIF plays a central role in neural control both at implantation and in the first trimester. Finally, the use of EGF in both cell types demonstrated that PIF’s effect is specific and can be blocked by the growth factor.

Overall, the current investigation reveals that the embryo, through PIF, conditions the maternal environment, specifically by targeting elements critical for survival (principally, the neural system). Hence, protection observed in both the newborn and the adult may be related to PIF’s role in neural development and protection that initiates in the earliest stages of pregnancy. Such beneficial maternal conditioning may have long-term ramifications in protecting against childhood and adult neurologic diseases.

## Competing interests

This work was supported through an unrestricted grant by BioIncept, LLC (MJP) and National Institutes of Health (grant number 5R01HD056123-02) (SJH). All other authors declare no competing interests.

## Authors’ contributions

CMD, MJP, and JSH carried out the experiments and helped in data analysis. LJ analyzed the MetaCore program. ERB discovered and developed the PIF technology, helped in data analysis, and wrote the manuscript with CMD, MJP, and LJ. All authors read and approved the final manuscript.

## Supplementary Material

Additional file 1 **Compare experiments workflow 1.0 data analysis report.** Data describes a detailed comparison and analysis of PIF effect on the HESC and FTDC gene data. This provides visual data of pathways and interactors present in the two time points. This also highlight similarities and differences between the two critical time points of embryo development, implantation, and embryogenesis. Click here for file

Additional file 2 **PIF effect on HESC global proteome in comparison with vehicle-treated control.** HESC were exposed to PIF for 24 h. Subsequently, the cells were collected, washed, and media extracted, and proteins were analyzed by semi-quantitative mass spectrometry. Data was analyzed by comparing PIF treated vs. control, where *p* < 0.05 was considered statistically significant. Click here for file
