# Supplementary material for: PreImplantation factor (PIF*) promotes embryotrophic and neuroprotective decidual genes: effect negated by epidermal growth factor
Source: J Neurodev Disord. 2014 Sep 11;6(1):36. doi: 10.1186/1866-1955-6-36 (PMC4470351; doi:10.1186/1866-1955-6-36)
Supplement: Additional file 1 — Compare experiments workflow 1.0 data analysis report. Data describes a detailed comparison and analysis of PIF effect on the HESC and FTDC gene data. This provides visual data of pathways and interactors present in the two time points. This also highlight similarities and differences between the two critical time points of embryo development, implantation, and embryogenesis. [file 1866-1955-6-36-S1.pdf]

Compare Experiments Workflow 1.0 Data Analysis Report

Server: *portal.genego.com*
Date: *2010-04-18*
Name: *Lellean JeBailey - GeneGo*
Login: *ljprojects*

Experiments

1.		HESC up and down-Fx pathway
2.		FTDC up and down-Fx pathway

Figure 1. The experiments uploaded for comparative analysis

Contact: Julie Bryant GeneGO, Inc. 169 Saxony Rd., #104, Encinitas CA, 92024
Phone: (858) 756 7996 E-mail: julie@genego.com

Table of content:

- Analysis settings
- Experiments comparison
- Enrichment analysis
  - GeneGo Pathway Maps
    - Top maps (sorted by common)
      - 1. Map : Muscle contraction_ GPCRs in the regulation of smooth muscle tone
      - 2. Map : Development_Angiotensin signaling via STATs
      - 3. Map : Development_Angiotensin activation of ERK
      - 4. Map : Development_ACM2 and ACM4 activation of ERK
      - 5. Map : Development_Angiotensin signaling via PYK2
  - GeneGo Process Networks
  - GO Processes
  - GeneGo Diseases (by Biomarkers)
- Most relevant networks
  - Top scored networks for common
    - Network 1
    - Network 2
    - Network 3
    - Network 4
    - Network 5
  - Top scored networks for Unique for HESC up and down-Fx pathway
    - Network 1
    - Network 2
    - Network 3
  - Top scored networks for Unique for FTDC up and down-Fx pathway
    - Network 1
    - Network 2
    - Network 3

Contact: Julie Bryant GeneGO, Inc. 169 Saxony Rd., #104, Encinitas CA, 92024
Phone: (858) 756 7996 E-mail: julie@genego.com

Analysis settings

(TOC)

Threshold	0
P-value threshold	1
Signals	both

Experiments comparison

(TOC)

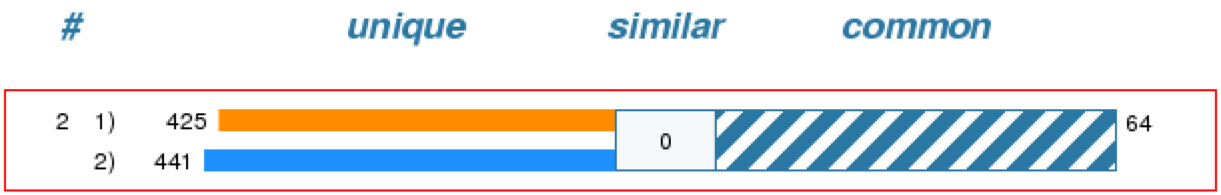

Figure 2. The gene content is aligned between all uploaded experiments. The intersection set of genes is defined as 'common' and marked as a blue/white striped bar. The unique genes for the experiments are marked as colored bars. The genes from the 'similar' set are present in all but one (any) file. The parameters for comparison are set as above.

Contact: Julie Bryant GeneGO, Inc. 169 Saxony Rd., #104, Encinitas CA, 92024
Phone: (858) 756 7996 E-mail: julie@genego.com

Enrichment analysis

(TOC)

Enrichment analysis consists of matching gene IDs of possible targets for the "common", "similar" and "unique" sets with gene IDs in functional ontologies in MetaCore. The probability of a random intersection between a set of IDs the size of target list with ontology entities is estimated in p-value of hypergeometric intersection. The lower p-value means higher relevance of the entity to the dataset, which shows in higher rating for the entity. The ontologies include GeneGo Pathway Maps, GeneGo Process Networks, GO Processes, GeneGo Diseases (by Biomarkers). The degree of relevance to different categories for the uploaded datasets is defined by p-values, so that the lower p-value gets higher priority.

GeneGo Pathway Maps

(TOC)

Canonical pathway maps represent a set of about 650 signaling and metabolic maps covering human biology (signaling and metabolism) in a comprehensive way. All maps are drawn from scratch by GeneGo annotators and manually curated & edited. Experimental data is visualized on the maps as blue (for downregulation) and red (upregulation) histograms. The height of the histogram corresponds to the relative expression value for a particular gene/protein.

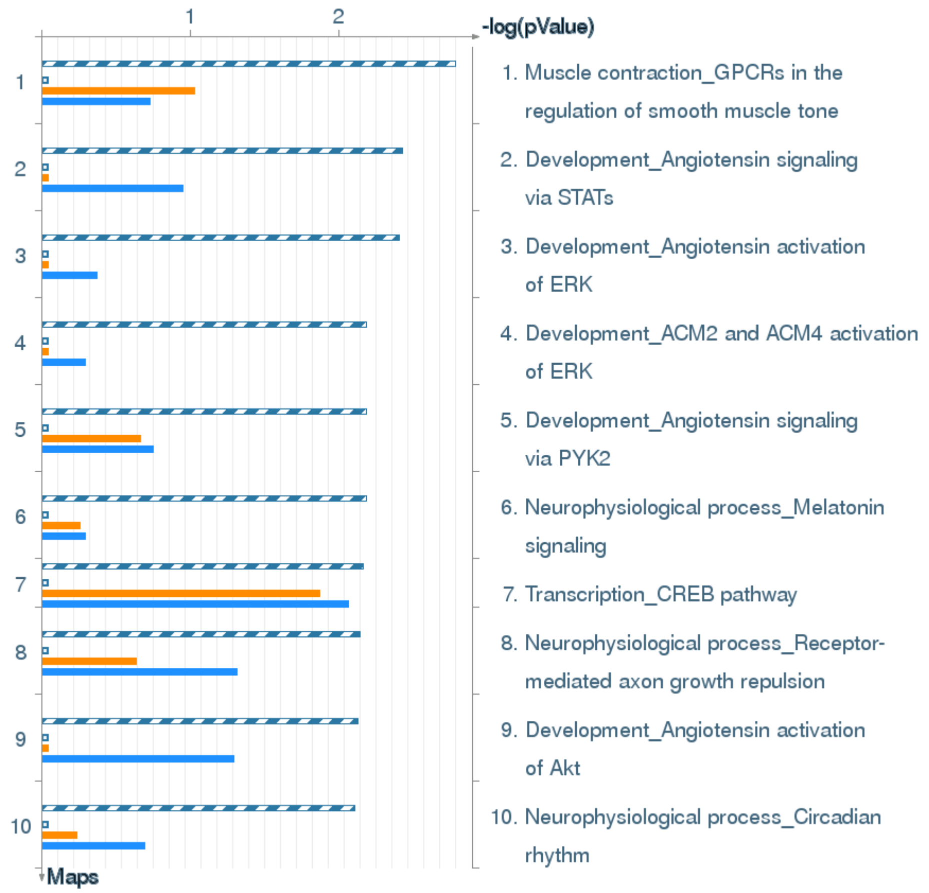

Figure 3. GeneGo Pathway Maps. Sorting is done for the 'common' set.

Contact: Julie Bryant GeneGO, Inc. 169 Saxony Rd., #104, Encinitas CA, 92024
Phone: (858) 756 7996 E-mail: julie@genego.com

Top maps (sorted by common)

1. Map : Muscle contraction_ GPCRs in the regulation of smooth muscle tone

(TOC)

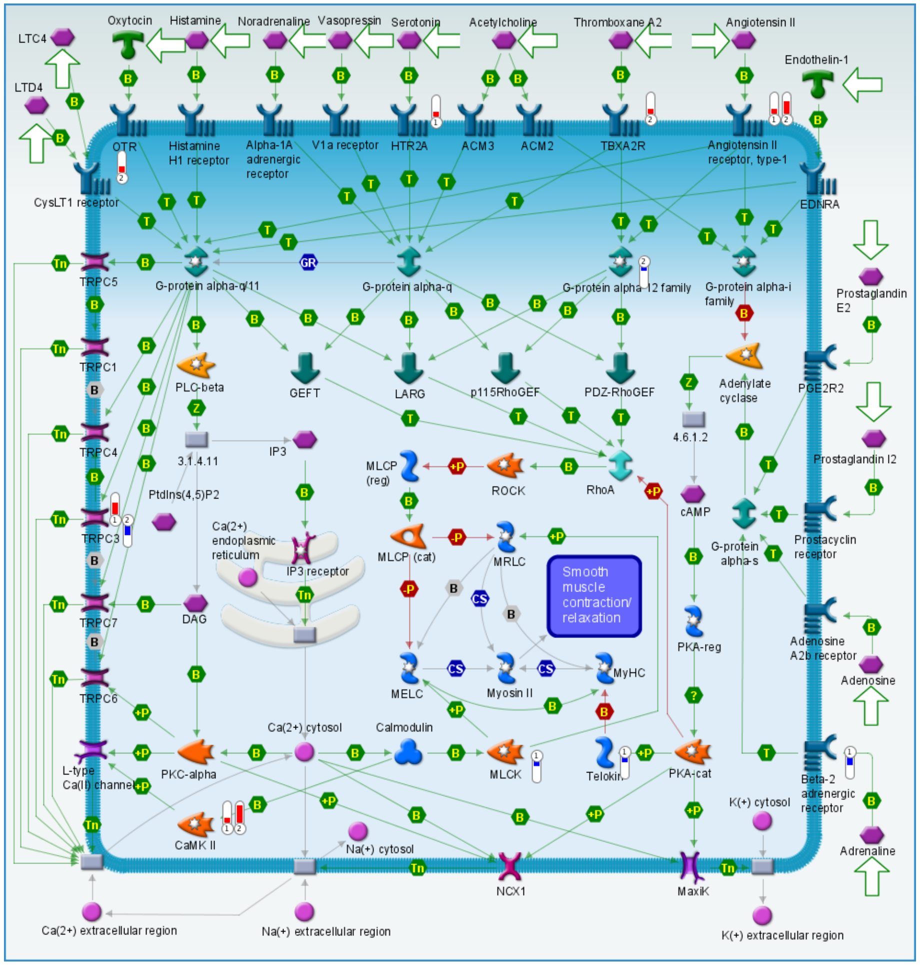

Figure 4. The top scored map (map with the the lowest p-value) based on the enrichment distribution sorted by 'common' set. Experimental data from all files is linked to and visualized on the maps as thermometer-like figures. Up-ward thermometers have red

Contact: Julie Bryant GeneGO, Inc. 169 Saxony Rd., #104, Encinitas CA, 92024
Phone: (858) 756 7996 E-mail: julie@genego.com

color and indicate up-regulated signals and down-ward (blue) ones indicate down-regulated expression levels of the genes.

Contact: Julie Bryant GeneGO, Inc. 169 Saxony Rd., #104, Encinitas CA, 92024
Phone: (858) 756 7996 E-mail: julie@genego.com

2. Map : Development_Angiotensin signaling via STATs

(TOC)

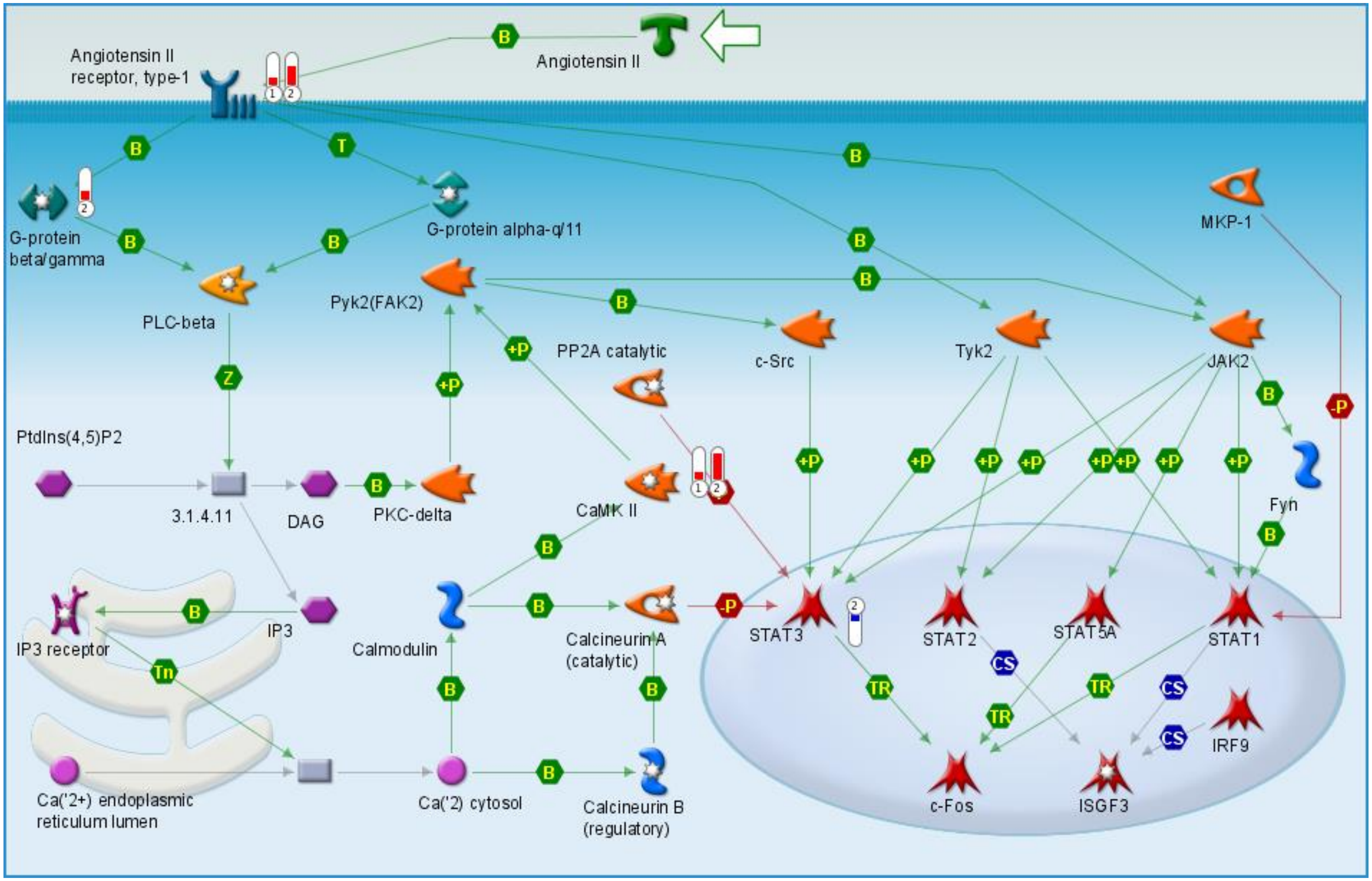

Figure 5. The second scored map (map with the second lowest p-value) based on the enrichment distribution sorted by 'common' set. Experimental data from all files is linked to and visualized on the maps as thermometer-like figures. Up-ward thermometers have red

color and indicate up-regulated signals and down-ward (blue) ones indicate down-regulated expression levels of the genes.

Contact: Julie Bryant GeneGO, Inc. 169 Saxony Rd., #104, Encinitas CA, 92024
Phone: (858) 756 7996 E-mail: julie@genego.com

3. Map : Development_Angiotensin activation of ERK

(TOC)

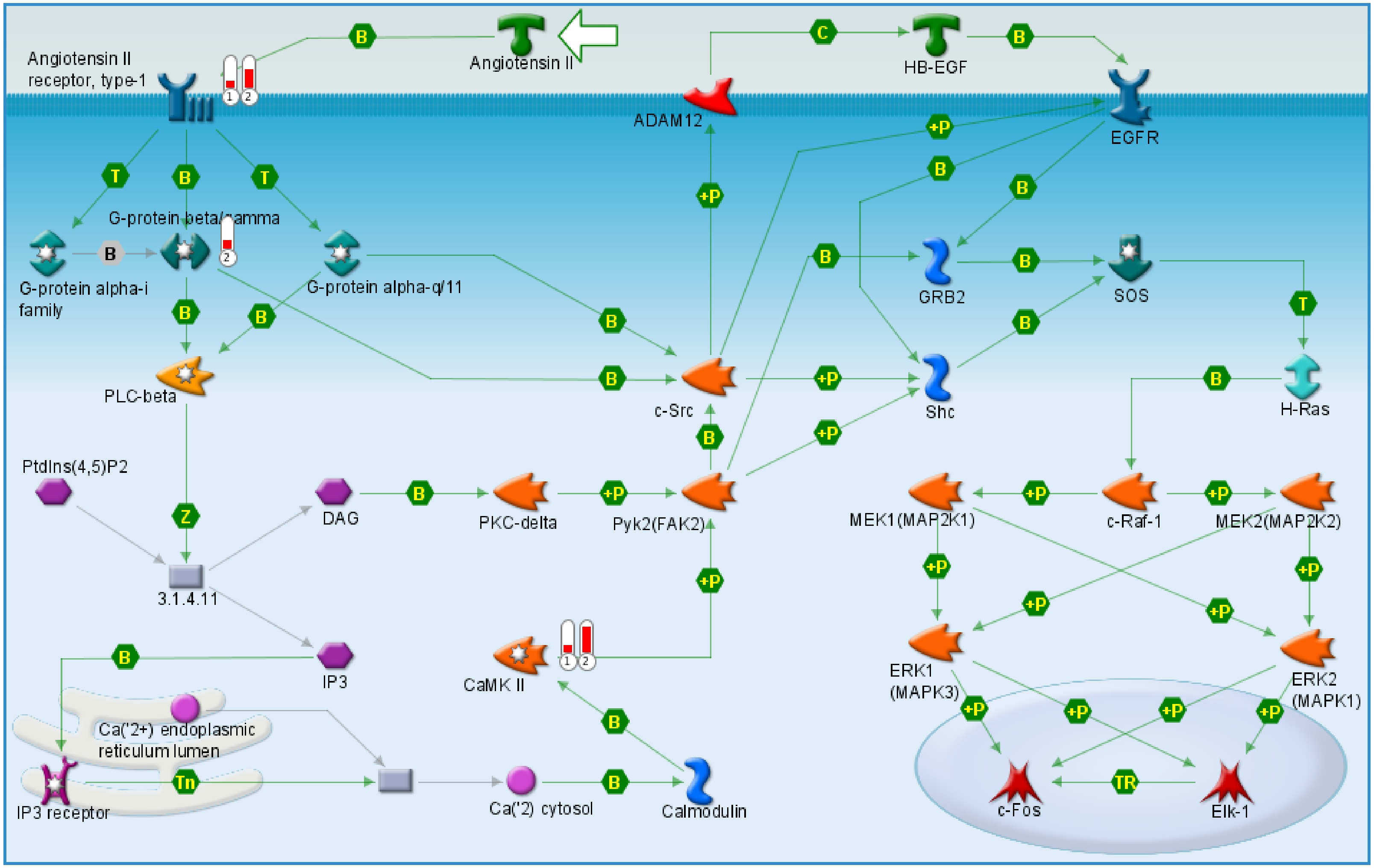

Figure 6. The third scored map (map with the third lowest p-value) based on the enrichment distribution sorted by 'common' set. Experimental data from all files is linked to and visualized on the maps as thermometer-like figures. Up-ward thermometers have red

color and indicate up-regulated signals and down-ward (blue) ones indicate down-regulated expression levels of the genes.

Contact: Julie Bryant GeneGO, Inc. 169 Saxony Rd., #104, Encinitas CA, 92024
Phone: (858) 756 7996 E-mail: julie@genego.com

4. Map : Development_ACM2 and ACM4 activation of ERK

(TOC)

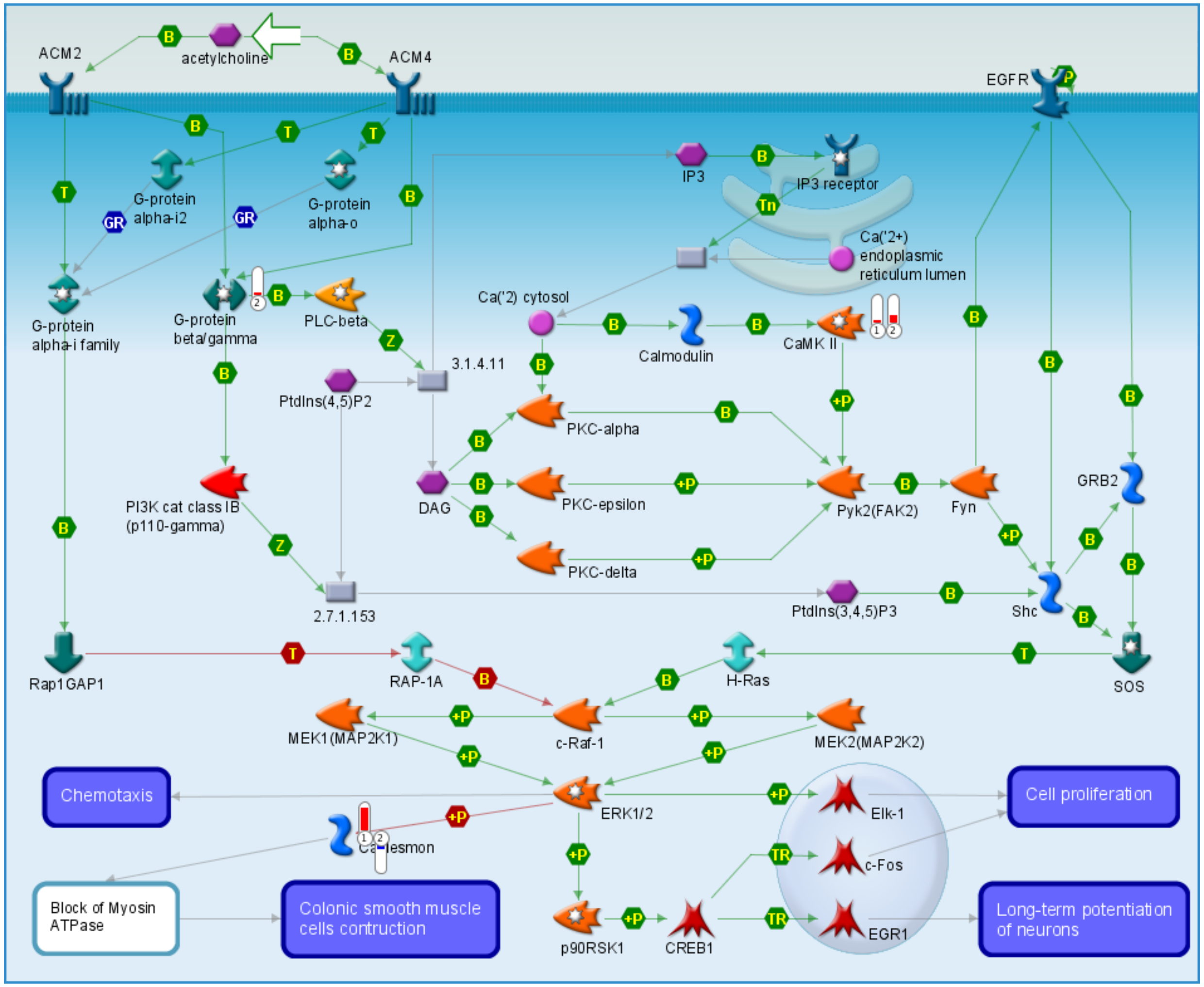

Figure 7. The fourth scored map (map with the fourth lowest p-value) based on the enrichment distribution sorted by 'common' set. Experimental data from all files is linked to and visualized on the maps as thermometer-like figures. Up-ward thermometers have red

color and indicate up-regulated signals and down-ward (blue) ones indicate down-regulated expression levels of the genes.

5. Map : [Development_Angiotensin signaling via PYK2](#)

(TOC)

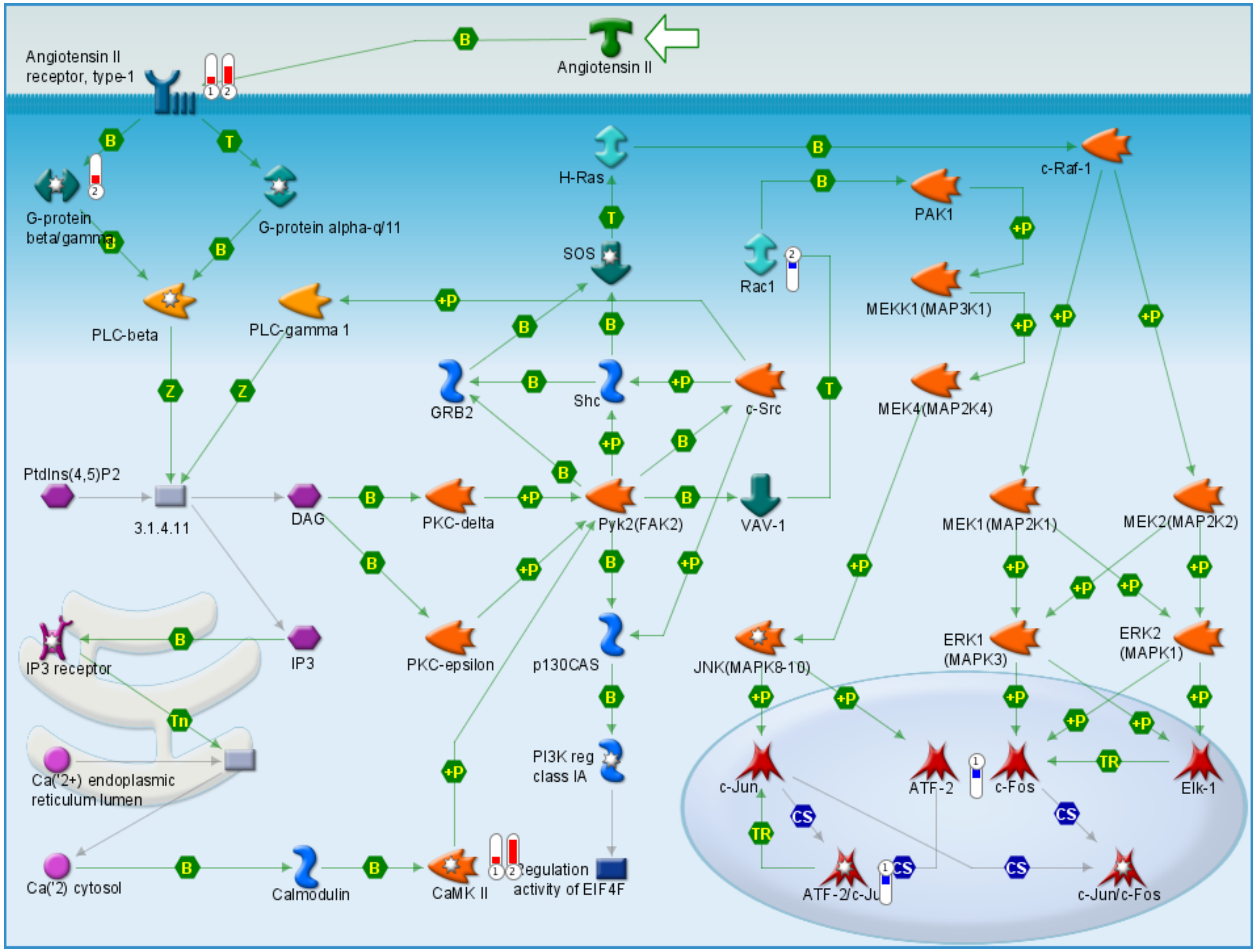

Figure 8. The fifth scored map (map with the fifth lowest p-value) based on the enrichment distribution sorted by 'common' set. Experimental data from all files is linked to and visualized on the maps as thermometer-like figures. Up-ward thermometers have red

color and indicate up-regulated signals and down-ward (blue) ones indicate down-regulated expression levels of the genes.

GeneGo Process Networks

(TOC)

These are about 110 cellular and molecular processes whose content is defined and annotated by GeneGo. Each process represents a pre-set network of protein interactions characteristic for the process. Experimental data is mapped on the processes and shown as red (up-regulation) and blue (down-regulation) circles of different intensity. Relative intensity corresponds to the expression value.

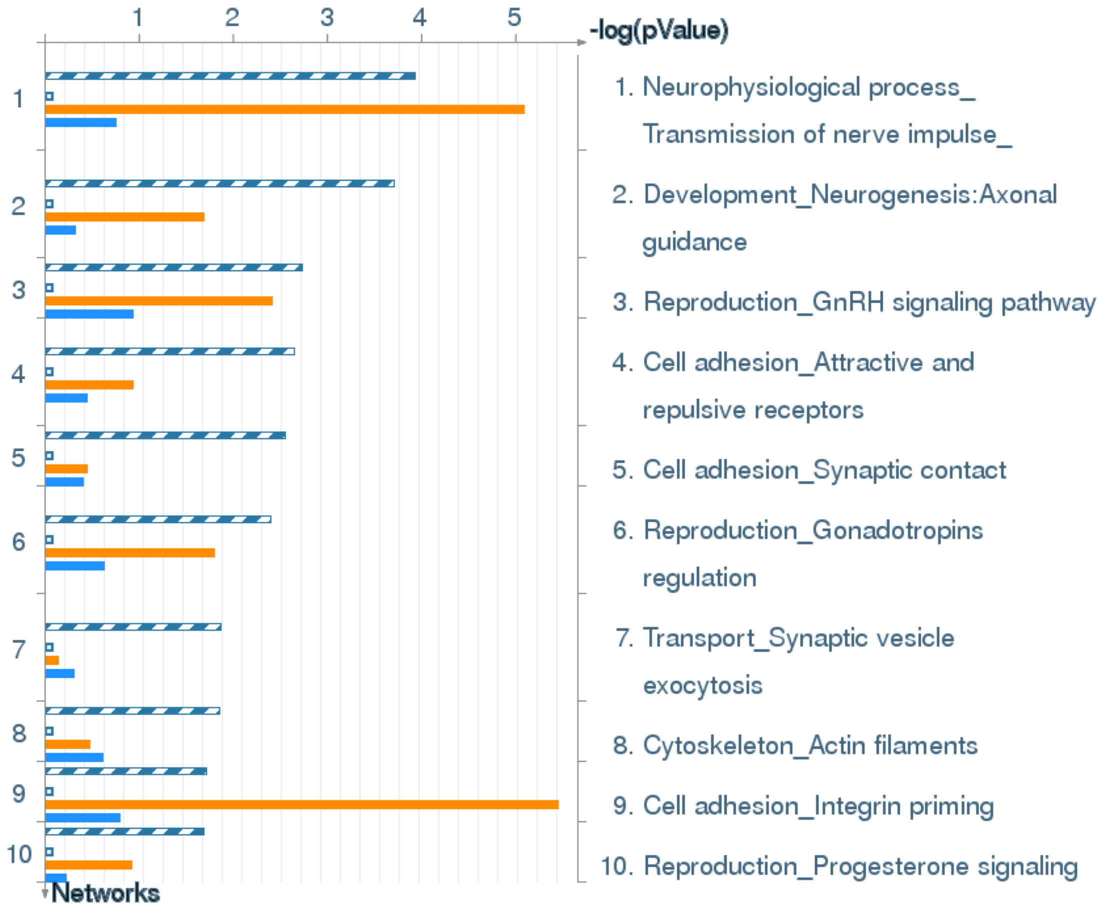

Figure 9. GeneGo Process Networks. Sorting is done for the 'common' set.

Contact: Julie Bryant GeneGO, Inc. 169 Saxony Rd., #104, Encinitas CA, 92024
Phone: (858) 756 7996 E-mail: julie@genego.com

GO Processes

(TOC)

These are the original Gene Ontology (GO) cellular processes, represented at GeneGo. Since most of GO processes have no gene/protein content, the "empty terms" are excluded from p-value calculations.

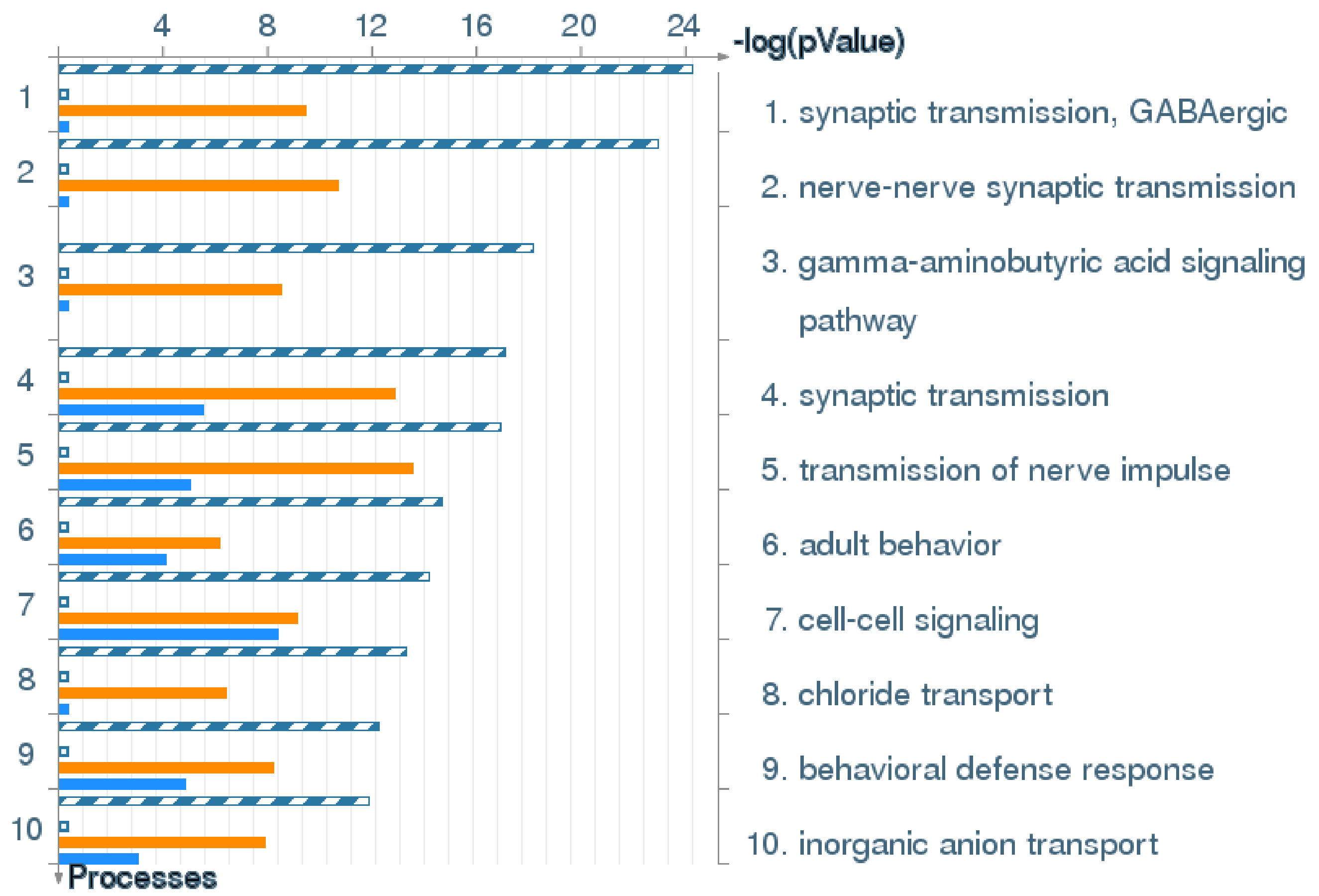

Figure 10. GO Processes. Sorting is done for the 'common' set.

Contact: Julie Bryant GeneGO, Inc. 169 Saxony Rd., #104, Encinitas CA, 92024
Phone: (858) 756 7996 E-mail: julie@genego.com

GeneGo Diseases (by Biomarkers)

(TOC)

Disease folders represent over 500 human diseases with gene content annotated by GeneGo. Disease folders are organized into a hierarchical tree. Gene content may very greatly between such complex diseases as cancers and some Mendelian diseases. Also, coverage of different diseases in literature is skewed. These two factors may affect p-value prioritization for diseases.

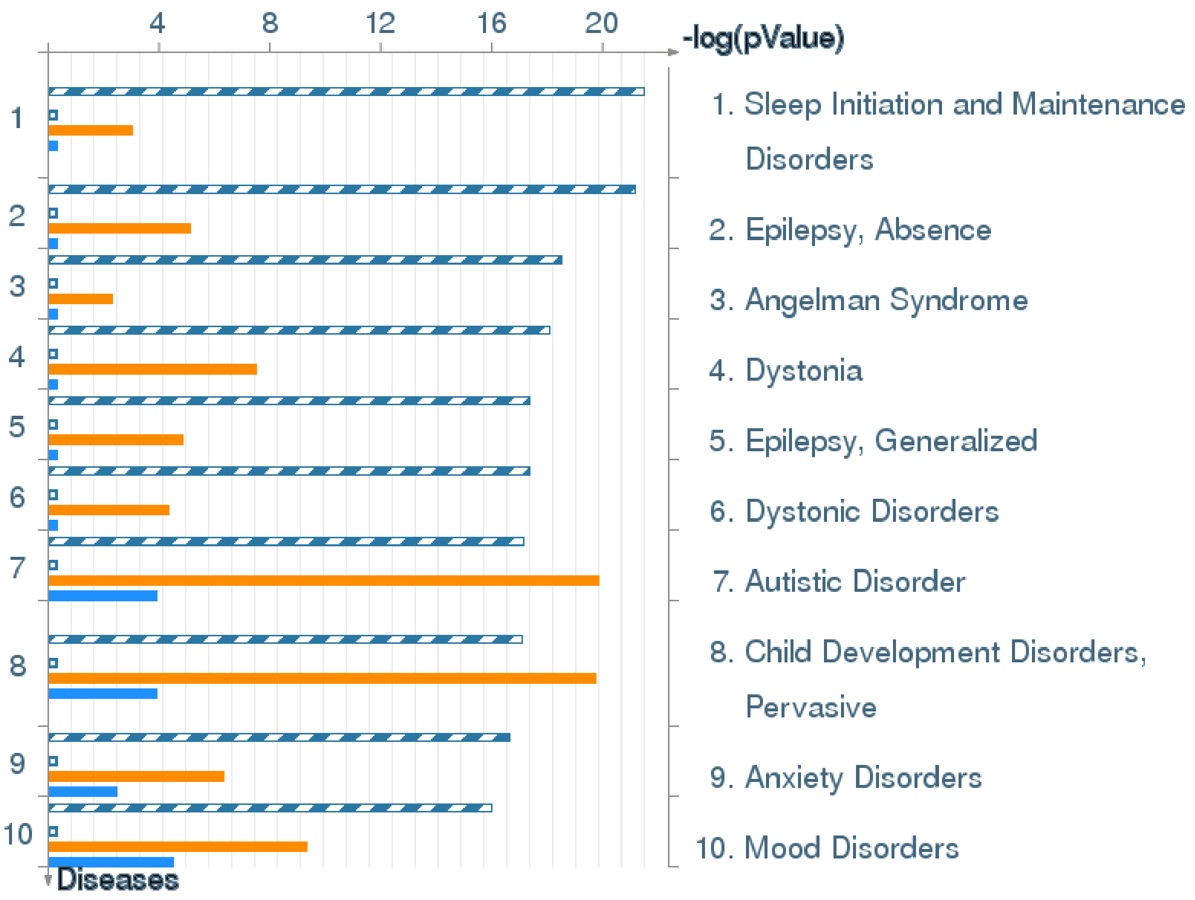

Figure 11. GeneGo Diseases (by Biomarkers). Sorting is done for the 'common' set.

Most relevant networks

(TOC)

The gene content of the uploaded files is used as the input list for generation of biological networks using Analyze Networks (AN) algorithm with default settings. This is a variant of the shortest paths algorithm with main parameters of 1. relative enrichment with the uploaded data, and 2. relative saturation of networks with canonical pathways. These networks are built on the fly and unique for the uploaded data. In this workflow the networks are prioritized based on the number of fragments of canonical pathways on the network.

No	Processes	Size	Target	Pathways	p-Value	zScore	gScore
	**common**						
1	generation of neurons (50.0%), neurogenesis (50.0%), nervous system development (60.4%)	50	8	26	8.93e-16	33.39	65.89
2	positive regulation of biological process (58.0%), response to steroid hormone stimulus (26.0%), positive regulation of cellular process (54.0%)	50	12	0	1.75e-25	50.20	50.20
3	enzyme linked receptor protein signaling pathway (37.5%), protein amino acid phosphorylation (39.6%), cell communication (77.1%)	50	10	0	1.32e-20	42.22	42.22
4	calcium ion transport (22.2%), divalent metal ion transport (22.2%), di-, tri-valent inorganic cation transport (22.2%)	50	9	0	3.39e-18	37.98	37.98
5	selenocysteine incorporation (8.8%), translational readthrough (8.8%), DNA replication initiation (8.8%)	50	8	0	6.35e-14	31.14	31.14
	**similar**						
	**Unique for FTDC up and down-Fx pathway**						
1	regulation of cell proliferation (72.5%), intracellular signaling cascade (78.4%), positive regulation of cell proliferation (58.8%)	51	9	288	1.24e-10	14.21	374.21
2	organ development (57.4%), anatomical structure morphogenesis (46.8%), response to oxygen levels (23.4%)	50	18	30	3.40e-26	29.32	66.82
3	regulation of immune system process (47.9%), positive regulation of immune system process (41.7%), positive	50	17	25	2.63e-24	27.66	58.91

	regulation of biological process (77.1%)						
	**Unique for HESC up and down-Fx pathway**						
1	anatomical structure formation (71.7%), regulation of cell differentiation (54.3%), positive regulation of cellular process (78.3%)	50	11	315	4.20e-14	18.19	411.94
2	tube development (26.7%), anatomical structure formation (44.4%), anatomical structure morphogenesis (44.4%)	50	18	24	7.00e-27	30.48	60.48
3	cAMP metabolic process (22.5%), response to hormone stimulus (47.5%), response to endogenous stimulus (47.5%)	51	20	20	4.14e-31	34.31	59.31

Top scored networks for common

1.

([TOC]())

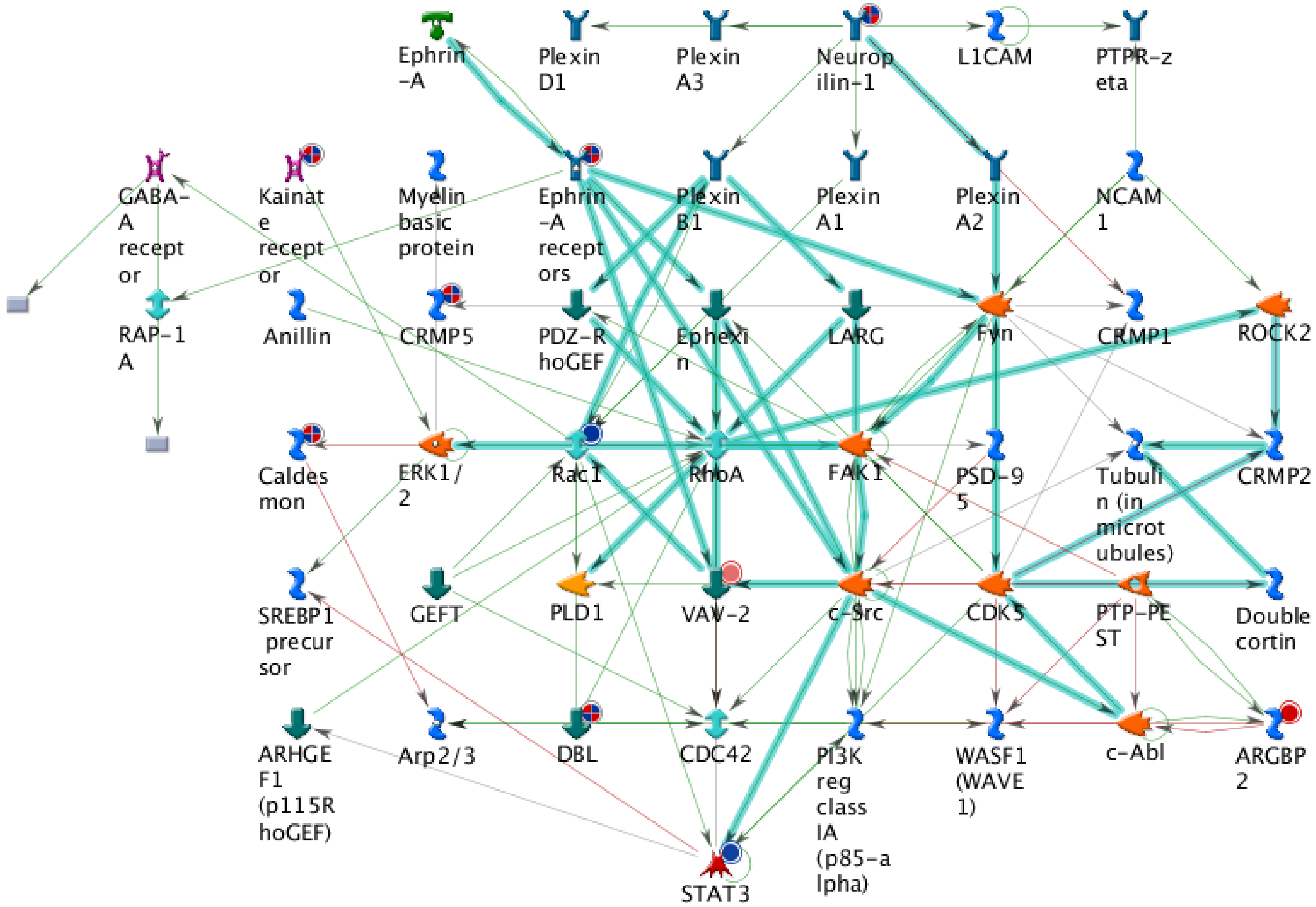

Figure 12. The top scored (by the number of pathways) AN network from common. Thick cyan lines indicate the fragments of

canonical pathways. Up-regulated genes are marked with red circles; down-regulated with blue circles. The 'checkerboard' color indicates mixed expression for the gene between files or between multiple tags for the same gene.

Contact: Julie Bryant GeneGO, Inc. 169 Saxony Rd., #104, Encinitas CA, 92024
Phone: (858) 756 7996 E-mail: julie@genego.com

2.

(TOC)

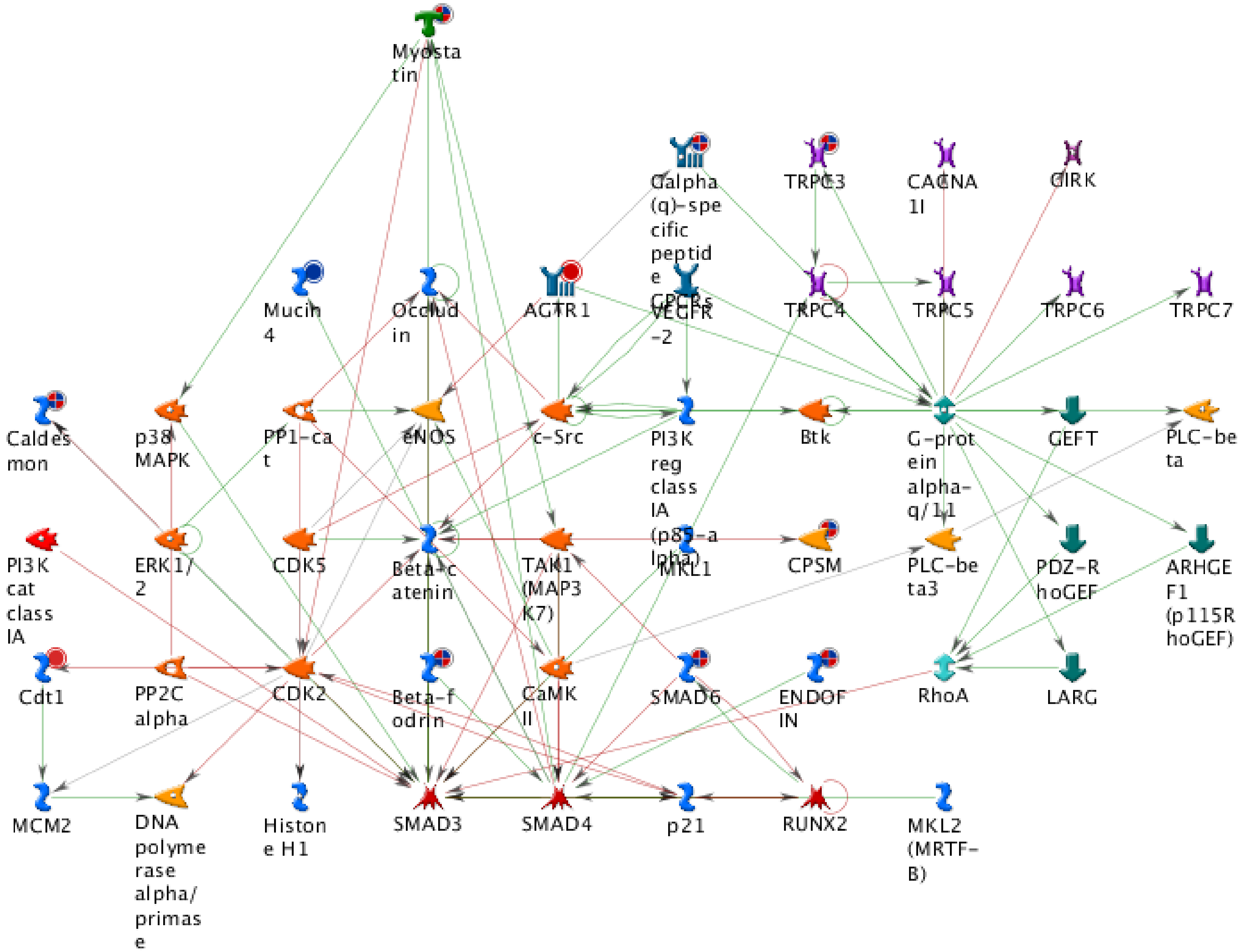

Figure 13. The second scored (by the number of pathways) AN network from common. Thick cyan lines indicate the fragments of canonical pathways. Up-regulated genes are marked with red circles; down-regulated with blue circles. The 'checkerboard' color

Contact: Julie Bryant GeneGO, Inc. 169 Saxony Rd., #104, Encinitas CA, 92024
Phone: (858) 756 7996 E-mail: julie@genego.com

indicates mixed expression for the gene between files or between multiple tags for the same gene.

Contact: Julie Bryant GeneGO, Inc. 169 Saxony Rd., #104, Encinitas CA, 92024
Phone: (858) 756 7996 E-mail: julie@genego.com

3.

(TOC)

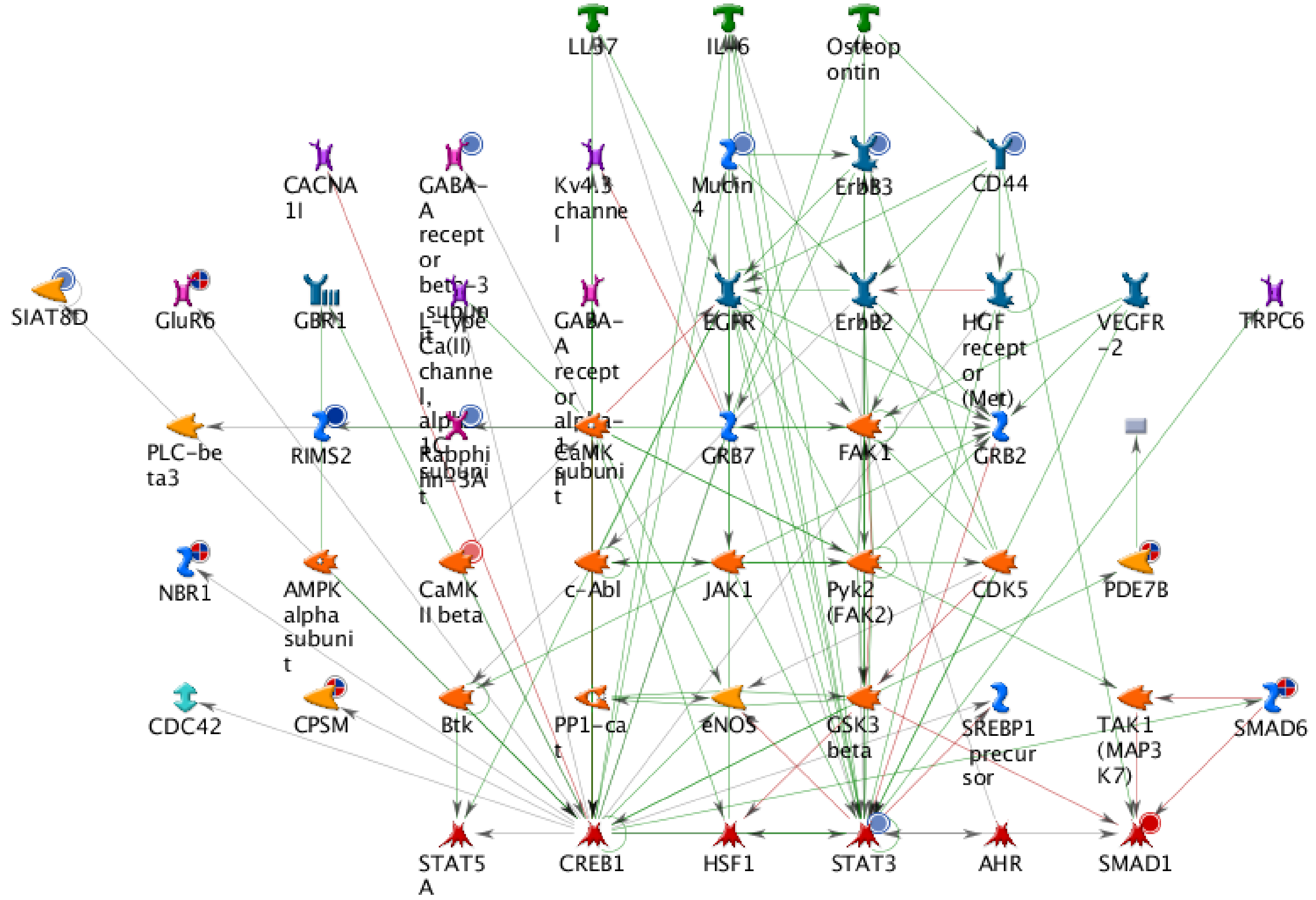

Figure 14. The third scored (by the number of pathways) AN network from common. Thick cyan lines indicate the fragments of canonical pathways. Up-regulated genes are marked with red circles; down-regulated with blue circles. The 'checkerboard' color

indicates mixed expression for the gene between files or between multiple tags for the same gene.

4.

(TOC)

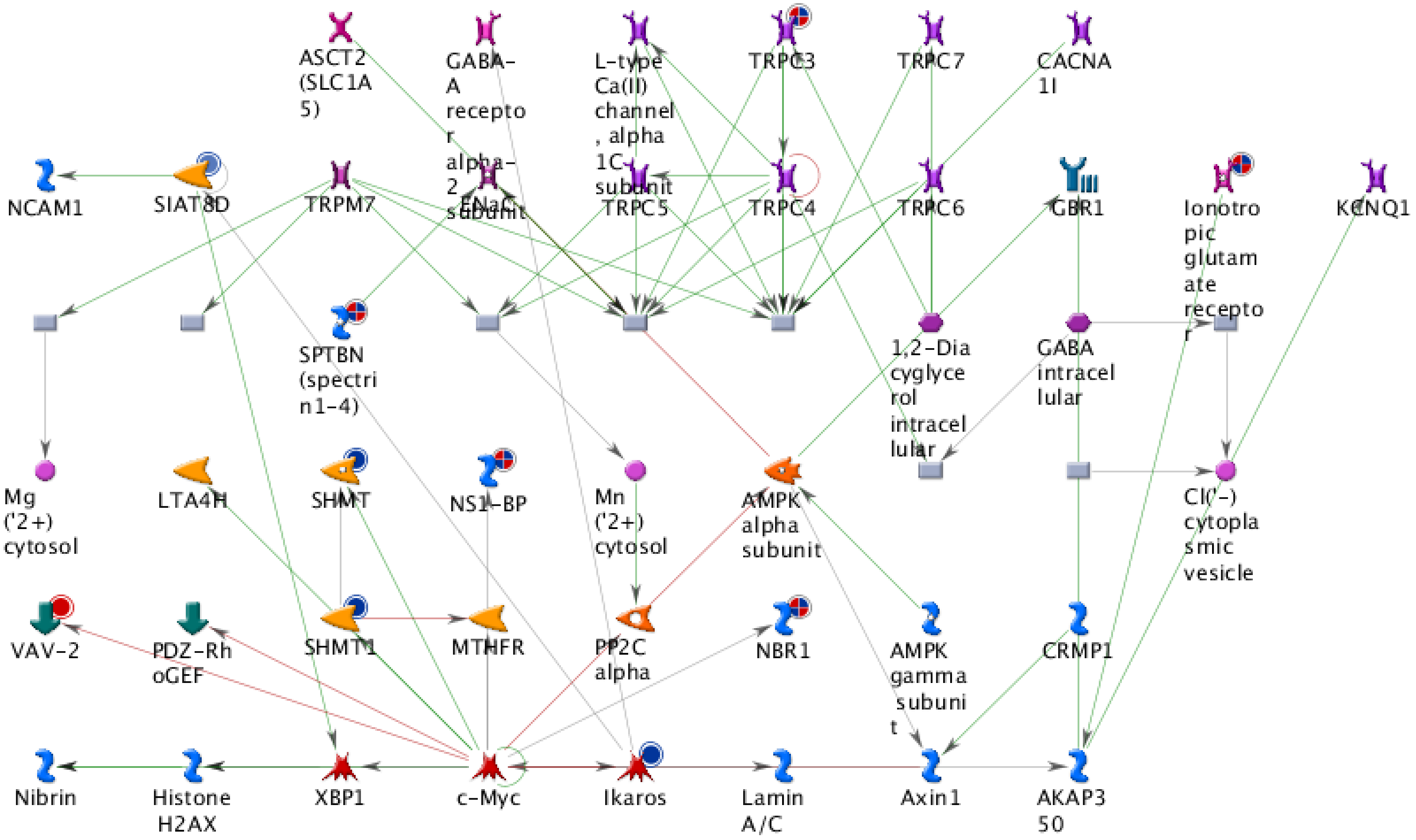

Figure 15. The fourth scored (by the number of pathways) AN network from common. Thick cyan lines indicate the fragments of canonical pathways. Up-regulated genes are marked with red circles; down-regulated with blue circles. The 'checkerboard' color indicates mixed expression for the gene between files or between multiple tags for the same gene.

5.

(TOC)

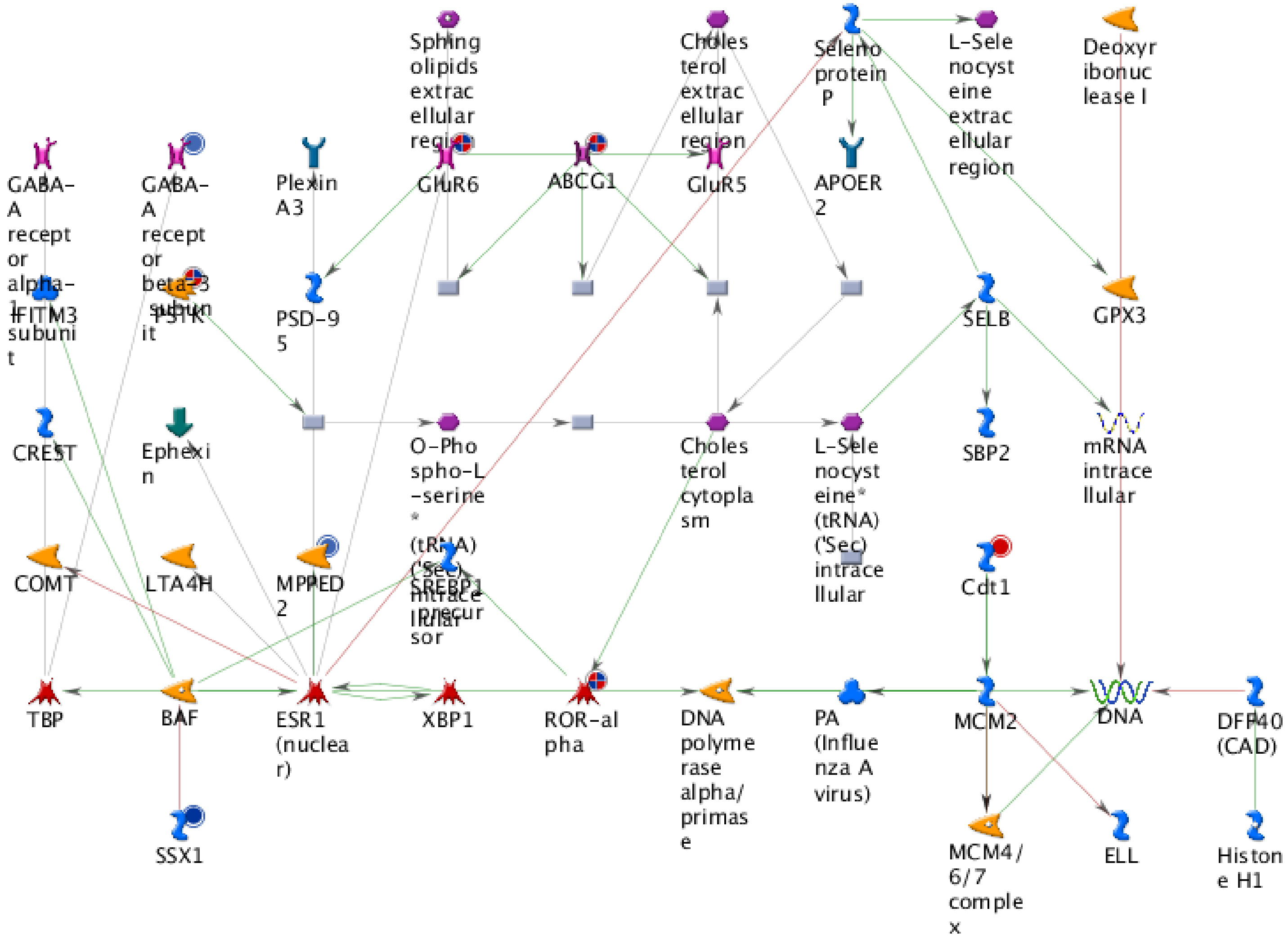

Figure 16. The fifth scored (by the number of pathways) AN network from common. Thick cyan lines indicate the fragments of canonical pathways. Up-regulated genes are marked with red circles; down-regulated with blue circles. The 'checkerboard' color

indicates mixed expression for the gene between files or between multiple tags for the same gene.

Top scored networks for Unique for HESC up and down-Fx pathway

1.

(TOC)

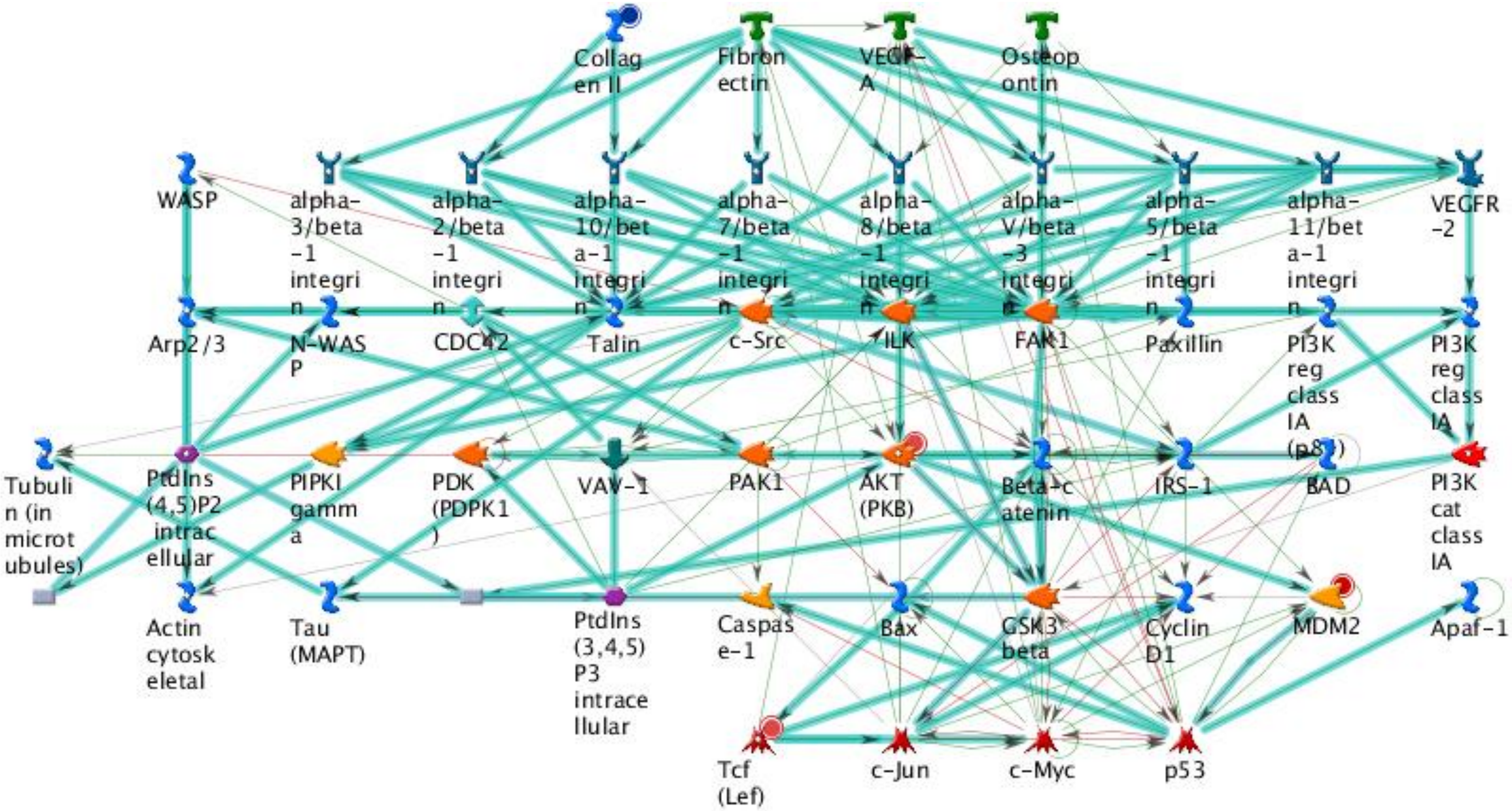

Figure 17. The top scored (by the number of pathways) AN network from Unique for HESC up and down-Fx pathway. Thick cyan lines indicate the fragments of canonical pathways. Up-regulated genes are marked with red circles; down-regulated with blue circles. The 'checkerboard' color indicates mixed expression for the gene between files or between multiple tags for the same gene.

2.

(TOC)

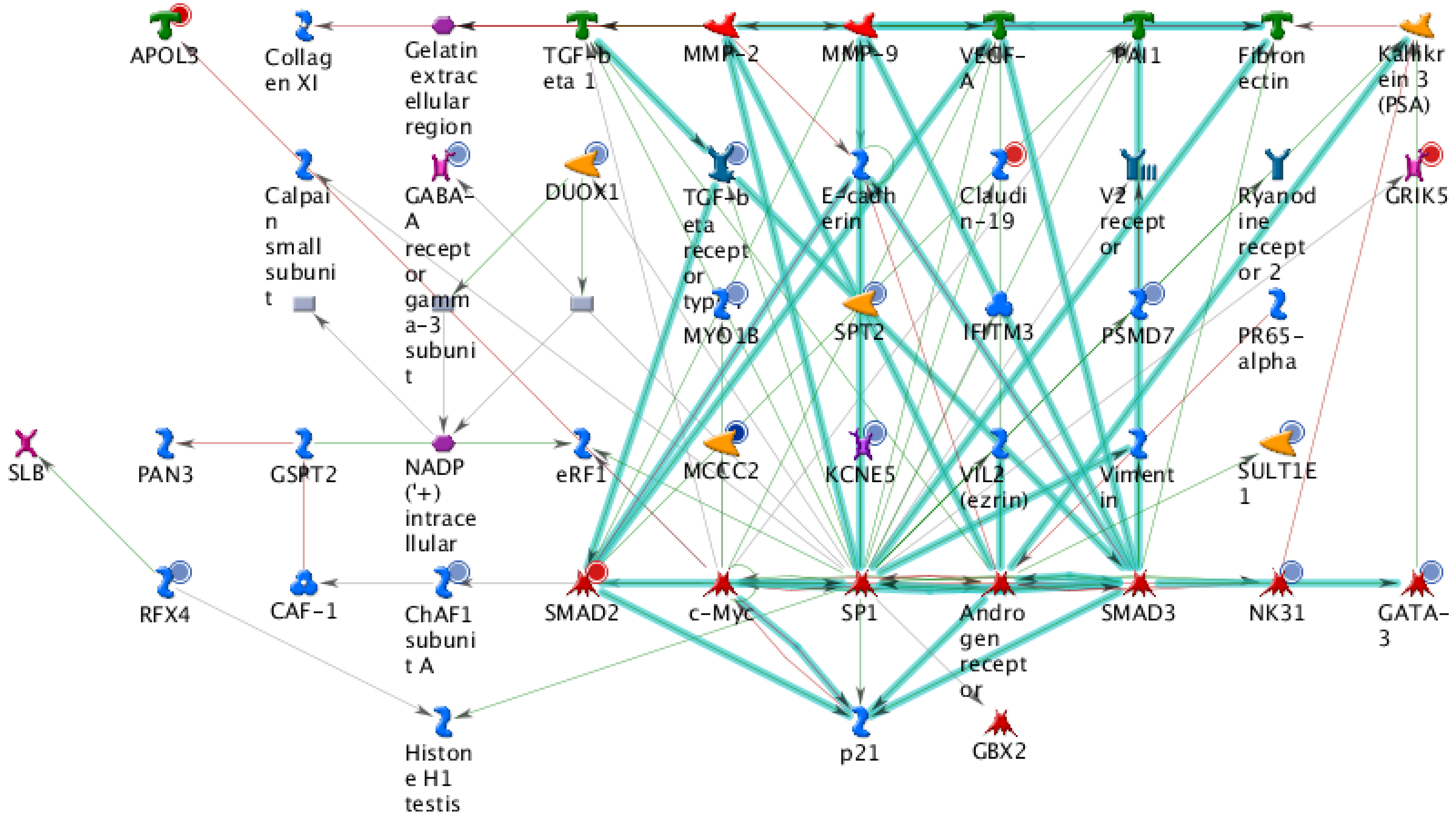

Figure 18. The second scored (by the number of pathways) AN network from Unique for HESC up and down-Fx pathway. Thick cyan lines indicate the fragments of canonical pathways. Up-regulated genes are marked with red circles; down-regulated with blue circles. The 'checkerboard' color indicates mixed expression for the gene between files or between multiple tags for the same gene.

3.

(TOC)

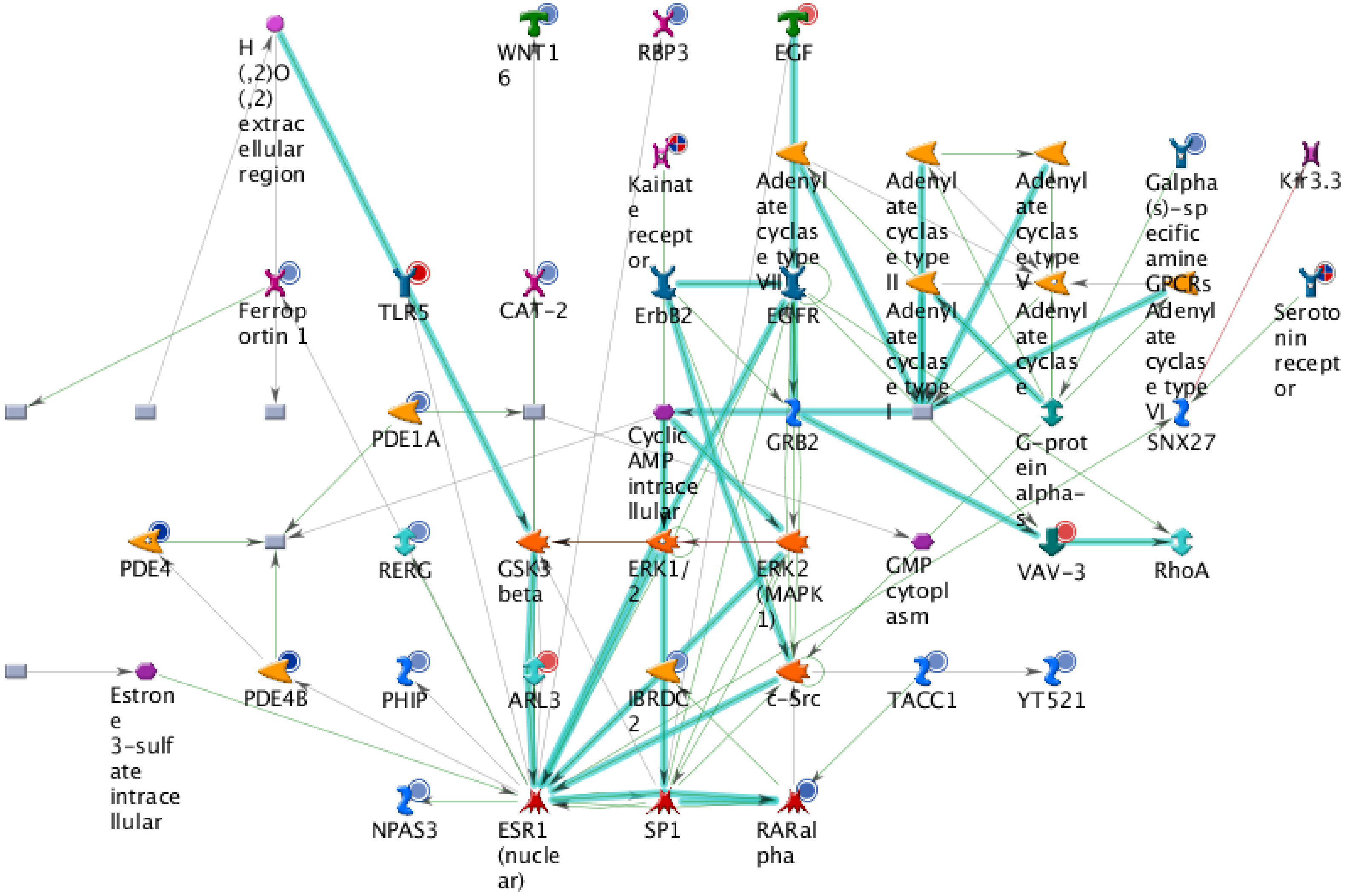

Figure 19. The third scored (by the number of pathways) AN network from Unique for HESC up and down-Fx pathway. Thick cyan lines indicate the fragments of canonical pathways. Up-regulated genes are marked with red circles; down-regulated with blue circles.

The 'checkerboard' color indicates mixed expression for the gene between files or between multiple tags for the same gene.

Contact: Julie Bryant GeneGO, Inc. 169 Saxony Rd., #104, Encinitas CA, 92024
Phone: (858) 756 7996 E-mail: julie@genego.com

Top scored networks for Unique for FTDC up and down-Fx pathway

1.

(TOC)

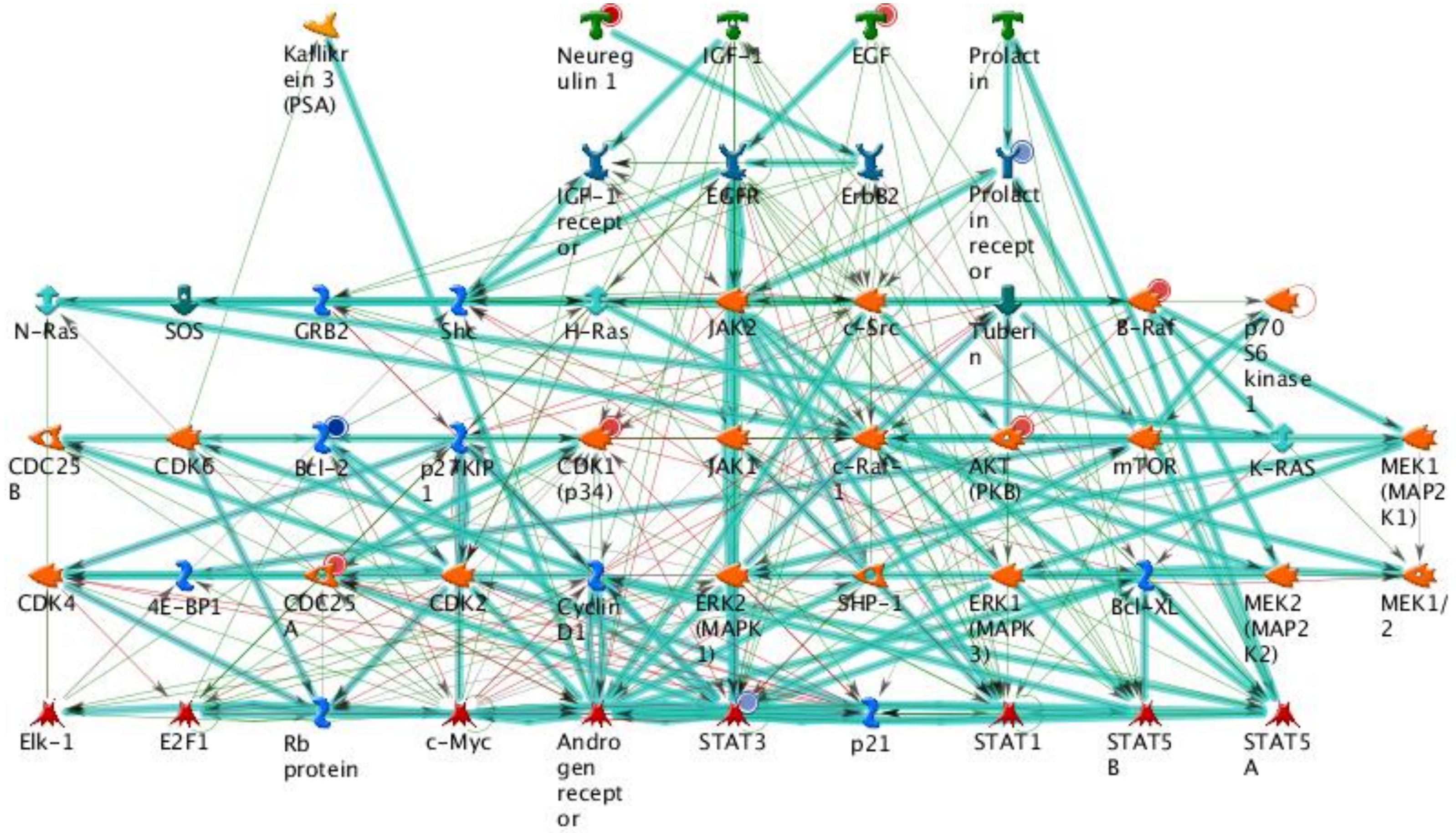

Figure 20. The top scored (by the number of pathways) AN network from Unique for FTDC up and down-Fx pathway. Thick cyan lines indicate the fragments of canonical pathways. Up-regulated genes are marked with red circles; down-regulated with blue circles. The 'checkerboard' color indicates mixed expression for the gene between files or between multiple tags for the same gene.

Contact: Julie Bryant GeneGO, Inc. 169 Saxony Rd., #104, Encinitas CA, 92024
Phone: (858) 756 7996 E-mail: julie@genego.com

2.

(TOC)

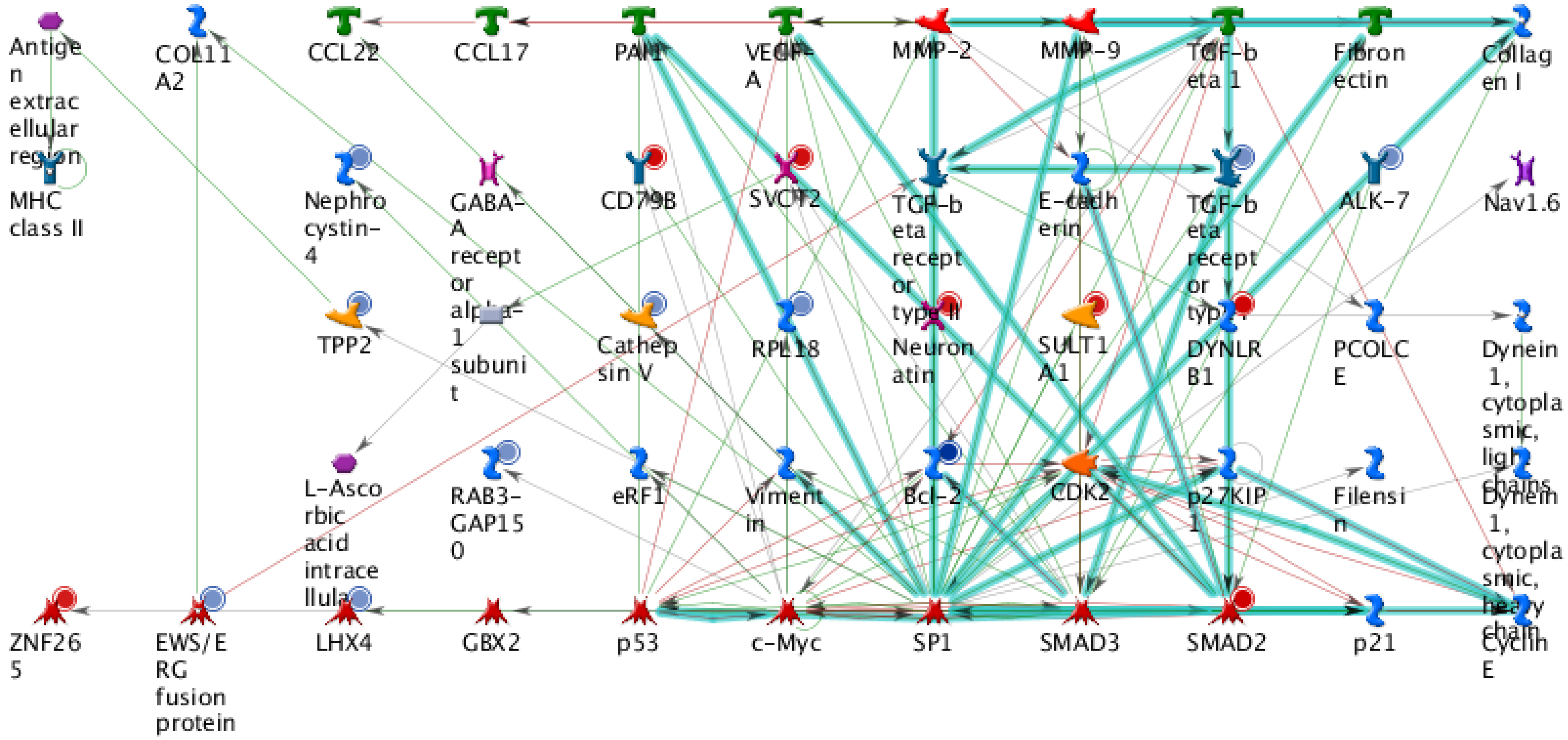

Figure 21. The second scored (by the number of pathways) AN network from Unique for FTDC up and down-Fx pathway. Thick cyan lines indicate the fragments of canonical pathways. Up-regulated genes are marked with red circles; down-regulated with blue circles. The 'checkerboard' color indicates mixed expression for the gene between files or between multiple tags for the same gene.

3.

(TOC)

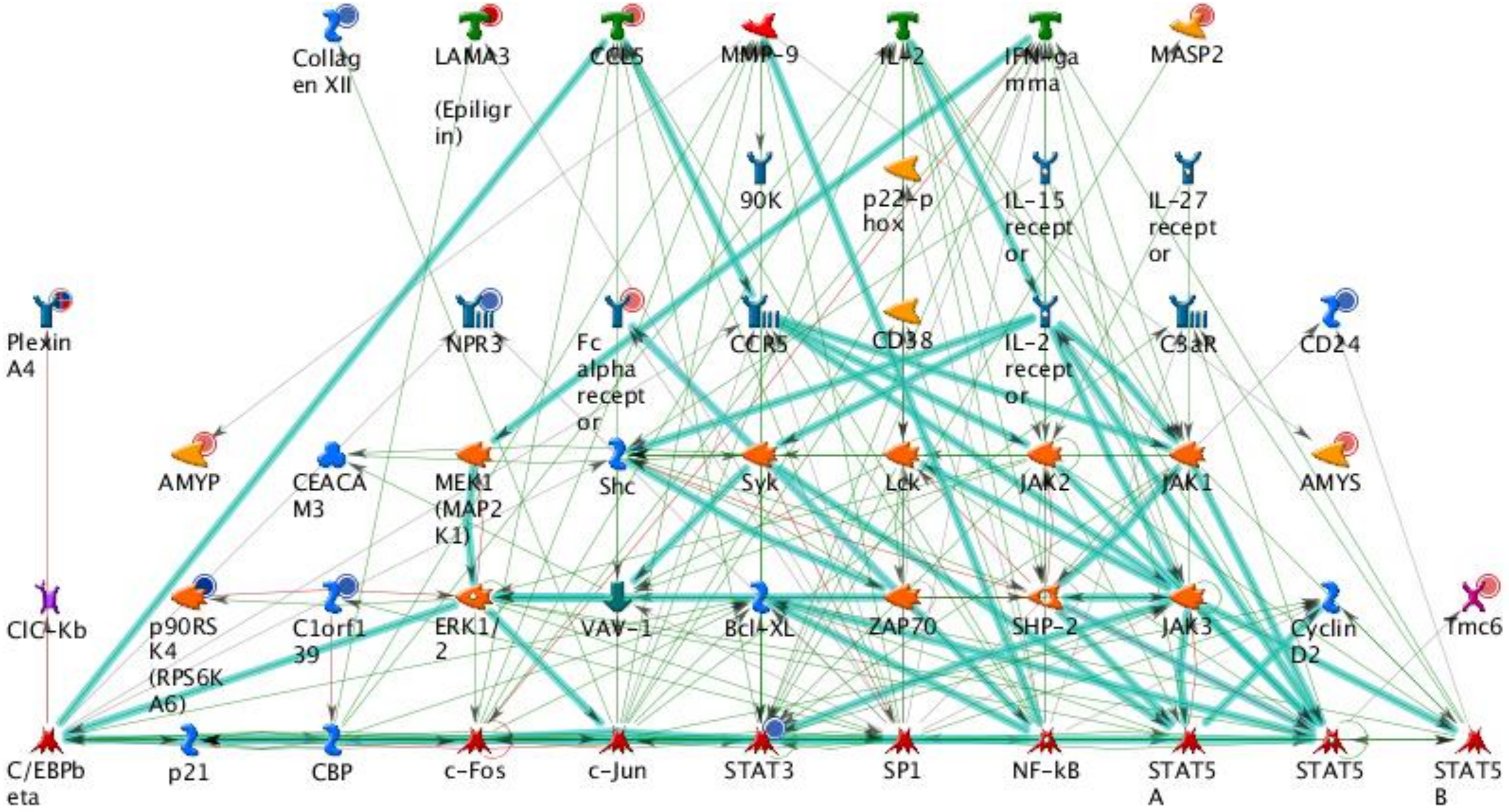

Figure 22. The third scored (by the number of pathways) AN network from Unique for FTDC up and down-Fx pathway. Thick cyan lines indicate the fragments of canonical pathways. Up-regulated genes are marked with red circles; down-regulated with blue circles. The 'checkerboard' color indicates mixed expression for the gene between files or between multiple tags for the same gene.

Contact: Julie Bryant GeneGO, Inc. 169 Saxony Rd., #104, Encinitas CA, 92024
Phone: (858) 756 7996 E-mail: julie@genego.com

Contact: Julie Bryant GeneGO, Inc. 169 Saxony Rd., #104, Encinitas CA, 92024
Phone: (858) 756 7996 E-mail: julie@genego.com
